# Computational Modeling to Quantify the Contributions of VEGFR1, VEGFR2, and Lateral Inhibition in Sprouting Angiogenesis

**DOI:** 10.3389/fphys.2019.00288

**Published:** 2019-03-27

**Authors:** Clemens Kühn, Sara Checa

**Affiliations:** ^1^Julius Wolff Institute, Charite - Universitätsmedizin Berlin, Berlin, Germany; ^2^Berlin-Brandenburg School for Regenerative Therapies, Charite - UIniversitätsmedizin Berlin, Berlin, Germany

**Keywords:** angiogenesis, VEGFR2, VEGFR1, lateral inhibition, agent based, computational model

## Abstract

Sprouting angiogenesis is a necessary process in regeneration and development as well as in tumorigenesis. VEGF-A is the main pro-angiogenic chemoattractant and it can bind to the decoy receptor VEGFR1 or to VEGFR2 to induce sprouting. Active sprout cells express Dll4, which binds to Notch1 on neighboring cells, in turn inhibiting VEGFR2 expression. It is known that the balance between VEGFR2 and VEGFR1 determines tip selection and network architecture, however the quantitative interrelationship of the receptors and their interrelated balances, also with relation to Dll4-Notch1 signaling, remains yet largely unknown. Here, we present an agent-based computer model of sprouting angiogenesis, integrating VEGFR1 and VEGFR2 in a detailed model of cellular signaling. Our model reproduces experimental data on VEGFR1 knockout. We show that soluble VEGFR1 improves the efficiency of angiogenesis by directing sprouts away from existing cells over a wide range of parameters. Our analysis unravels the relevance of the stability of the active notch intracellular domain as a dominating hub in this regulatory network. Our analysis quantitatively dissects the regulatory interactions in sprouting angiogenesis. Because we use a detailed model of intracellular signaling, the results of our analysis are directly linked to biological entities. We provide our computational model and simulation engine for integration in complementary modeling approaches.

## Introduction

Angiogenesis is a pivotal process in various aspects of vertebrate life. In development (Breier, 2000) as well as in regenerative processes like wound healing (Flegg et al., 2015) and bone fracture healing (Checa and Prendergast, 2009; Carlier et al., 2015a), angiogenesis is necessary to support newly forming tissue with oxygen and nutrients. Tumors abuse sprouting angiogenesis to direct vascularization toward them (MacGabhann and Popel, 2006). A quantitative understanding of the molecular mechanisms that shape vascular network structure can hence lead to improved treatment options in regenerative medicine and beyond, such as in oncology.

In sprouting angiogenesis, quiescent endothelial cells (ECs) in an existing vessel adopt a motile tip cell phenotype, release matrix metalloproteases (MMPs) to degrade the extracellular matrix (ECM) around the vessel, and lead a sprout followed by proliferative stalk cells (Logsdon et al., 2014). Eventually, the sprouts extend to neighboring vessels and undergo anastomosis and lumenization, followed by pruning and further maturation of the vessel network (Chappell et al., 2011; Tung et al., 2012). Direct cell-cell signaling and signaling via biochemical gradients establish different cell types in angiogenesis. Endothelial cells in emerging vessels can be roughly divided into three different phenotypes: motile tip cells that lead a vessel sprout, stalk cells that are proliferative but are not motile, and phalanx cells that are neither motile nor proliferative but form strong cell-cell junctions in maturating vessels (Carmeliet and Jain, 2011). The distinction between tip cells, stalk cells, and phalanx cells is usually achieved via imaging of different marker genes (Ubezio et al., 2016). Recent data show that the expression of these marker genes is highly dynamic and results in dynamic cell state changes (Venkatraman et al., 2016). Different microenvironments along a sprout can lead to the emergence of tip-like cells behind the sprout tip that eventually overtake the actual tip cell (Jakobsson et al., 2010). The dynamic expression patterns, on the single cell level, can also lead to oscillatory expression patterns of tip cell associated genes on the tissue level that modify the topology of the emerging network in developmental retinal angiogenesis (Ubezio et al., 2016).

VEGF-A induced Delta-Notch signaling drives tip cell selection (Liu et al., 2003; Suchting et al., 2007; Bentley et al., 2009). VEGF-A activates VEGF-receptor 2 (VEGFR2, also called Flk-1), which leads to the expression of tip cell markers and sprouting. VEGFR2 activity also leads to increased expression of *dll4*, activating Notch1 in neighboring cells through cleavage of the notch intracellular domain (NICD) which in turn represses *VEGFR2* expression (and subsequently tip cell markers and *dll4* expression) through regulation of transcription factors HES2 and HEY2 (Suchting et al., 2007). This lateral inhibition of tip cell activation induces patterns where tip cells are surrounded by non-tip cells (Hellström et al., 2007; Lobov et al., 2007; Venkatraman et al., 2016). VEGF/Delta-Notch signaling is not isolated, but a wide range of factors can modify tip cell selection (Harrington et al., 2008; Geudens and Gerhardt, 2011; Benn et al., 2017). Different splice variants of VEGF-A and different compositions of the ECM can lead to different diffusion and binding properties and thus influence sprouting (Keyt and Berleau, 1996; Ruhrberg et al., 2002; Ferrara et al., 2003). Additionally, different members of the VEGF family can interact with different receptors (Cao, 2009).

The decoy receptor VEGF-receptor 1 (VEGFR1, also called Flt-1) binds VEGF-A with a higher affinity than VEGFR2, however leads to negligible activation of downstream targets (Waltenberger et al., 1994). In spite of this, VEGFR1 plays an important role in angiogenesis, as disruption of VEGFR1 leads to embryonic lethality through vascular overgrowth (Fong et al., 1995). The presumed role of VEGFR1 in angiogenesis is to reduce VEGFR2 activity by reducing local VEGF-A availability (Hiratsuka et al., 1998; Roberts et al., 2004). A soluble splicing isoform, sVEGFR1, is secreted and can diffuse through extracellular space to reduce VEGF-A availability in a radius around a secreting cell (Kendall and Thomas, 1993; Roberts et al., 2004). Transcriptional regulation of *VEGFR1* is contrary to the regulation of *VEGFR2*, namely activated by Dll4-Notch1 signaling (Harrington et al., 2008; Funahashi et al., 2010) so that *VEGFR1* is mainly expressed in stalk cells and inhibited in tip cells while *VEGFR2* is a tip cell marker and suppressed in stalk cells. Accordingly, the general mechanisms by which VEGFR1 regulates angiogenesis have been identified, but their interdependencies with activatory VEGFR2 signaling and lateral inhibition via Delta-Notch are difficult to collectively assess experimentally.

VEGF-A has been implied as a target in several clinically relevant conditions, e.g., ischemia, arthritis, or obesity (Carmeliet, 2003), peripheral artery disease (Clegg et al., 2017), or cancer (MacGabhann and Popel, 2006; Zhang et al., 2010). Anti-VEGF drugs have been very successful in the treatment of age-related macula degeneration and diabetic macula oedema (Virgili et al., 2012; Solomon et al., 2014). To maximize the effectiveness of anti-VEGF drugs in cancer therapy, however, they need to be combined with drugs targeting different pathways (Jain et al., 2006; Carmeliet et al., 2009; Incio et al., 2018; Wagner et al., 2018). Optimization of such combinatorial therapies requires detailed knowledge of the dynamics within and between pathways. Computational studies can generate this knowledge efficiently and can also be used to predict optimal treatment regimens (Barros de Andrade e Sousa et al., 2016). Hence an improved understanding of the dynamic interplay of VEGF pathway components can contribute to improved treatments against a wide variety of conditions.

A considerable number of computational models of sprouting angiogenesis exist (Merks et al., 2004; Bentley et al., 2008, 2009; Jakobsson et al., 2010; Carlier et al., 2012, 2014, 2015b; van Oers et al., 2014; Boas and Merks, 2015; Heck et al., 2015; Walpole et al., 2015; Ubezio et al., 2016; Venkatraman et al., 2016; Bentley and Chakravartula, 2017). Most of these models are based on the “memAgent-Spring” (Bentley et al., 2008), Cellular Potts (Merks et al., 2004), or agent based (Carlier et al., 2014) modeling approaches.

The “memAgent-Spring Model” describes the alignment of membrane patches assigned to specific cells on a pre-defined shape, each patch can have its own dynamics concerning signaling (Bentley et al., 2008). Models using this approach have been used to explain, for example, tip cell overtaking (Jakobsson et al., 2010) and oscillations in lateral inhibition (Ubezio et al., 2016). Although this kind of model can be used to describe tip selection and intercellular signaling in a sprout in high detail, the restriction to a static shape on which the cells are aligned makes it impossible to simulate vascular network formation.

In Cellular Potts models, cells are described by a collection of nodes on a lattice. Cell shapes and movement arise dynamically from re-assigning nodes to minimize an energy function (Merks et al., 2004). This energy function can contain various terms, e.g., to constrain cell area or perimeter or bias movement to a certain direction. Cellular Potts models can describe network formation from individual cells (Köhn-Luque et al., 2013) and have also been used to explain tip cell overtaking (Boas and Merks, 2015). A drawback of Cellular Potts models is that terms of the energy function can be added arbitrarily and are often difficult to link to biological mechanisms. In addition, the computational cost renders simulations of vessel networks in 3D currently unpractical.

Agent based models in which single cells are represented by single nodes on a lattice have been used to describe blood vessel formation in bone healing scenarios (Checa and Prendergast, 2010; Carlier et al., 2015b). Although they omit dynamics of cellular shape, they can be used to simulate collective cellular organization (Checa et al., 2015) and computational complexity is low enough to permit simulation of network formation in 3D. Existing agent based models of angiogenesis ignore or strongly simplify biochemical signaling (Checa and Prendergast, 2010; Carlier et al., 2014, 2015b). In these models, the tip cell phenotype is either enforced (Checa and Prendergast, 2010) or emerges as a result of simple rules related to contact inhibition (Carlier et al., 2014, 2015b). In the latter, model predictions overestimate vessel growth.

Computational models have also been used to explore the role of VEGFR1 in angiogenesis. Most of these models focus on the establishment of VEGF-A and VEGF receptor gradients and binding kinetics (MacGabhann and Popel, 2004; Wu et al., 2010; Hashambhoy et al., 2011; Chappell et al., 2016). Walpole et al. (2015) described cellular movement, but not sprouting as a whole, in a Cellular Potts-like framework. To date, there is no computer model that integratively investigates the signaling interactions of VEGFR2, VEGFR1, and Delta-Notch during sprouting, their interdependencies and their effects on the forming vascular network.

Here, we present a computational agent based model to quantitatively dissect the interrelation of the regulatory mechanisms in tip selection and sprouting. We focus on Delta-Notch and VEGF signaling including VEGFR1 and VEGFR2. Our model allows us to assess the experimentally inaccessible interdependencies of intracellular signaling and extracellular conditions. Our analysis shows that VEGFR1 is efficient in guiding sprouts away from existing vessels and it also highlights the importance of Delta-Notch signaling, specifically the degradation of NICD, for angiogenesis. We provide our model in an open and reproducible way, thus facilitating integration into different contexts.

## Materials and Methods

###  Agent Based Model Simulator

We use a custom simulator, AngioABM, for our agent based modeling, implemented in C++11 using the boost libraries (The Boost Community, 2017). The source code is available under the Apache License 2.0 at https://gitlab.com/ModularABM/AngioABM/tree/VEGFR1. Scripts for visualization and analysis are available at https://gitlab.com/ModularABM/ABMTools/tree/VEGFR1.

In AngioABM, an agent based model consists of global variables, local variables and agents placed on a discrete 2D grid. Global variables are single numerical values defined for all positions of the grid. Local variables are 2D matrices of the same dimensions as the grid. Agents contain an id, coordinates, internal variables, and update rules. For clarity, we will refer to local variables using monospace font and to agents internal variables in *italics*. AngioABM reads model descriptions in XML.

**Figure 1:**
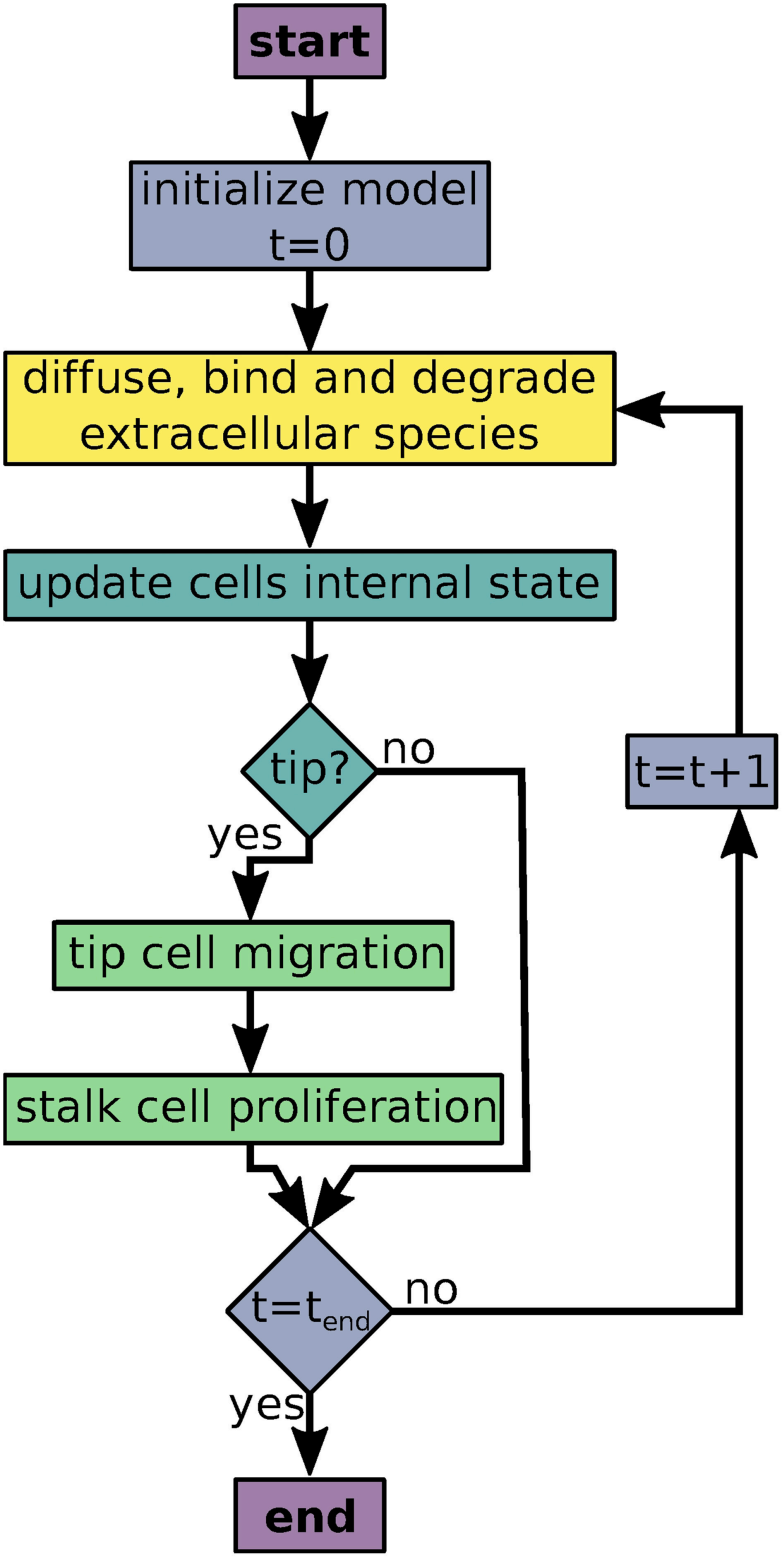
Flowchart of the agent based model simulations. Colors indicate processes/decisions associated with simulation time (blue), local variables (yellow), agents' intracellular dynamics (turquoise), and agent movement and proliferation (green).

AngioABM uses discrete time steps for time course simulations. At each time step, update and output functions of all local variables and all agents are called iteratively as shown in Figure 1.

Local variables represent molecules (in particle numbers per lattice site). Basic functions for diffusion, degradation, and adding molecules are implemented in a base class Localvar. These functions can be used to implement derived classes of local variables that exhibit custom dynamics. AngioABM uses forward integration to compute local variables' temporal and spatial evolution. The simulator assumes Dirichlet boundary conditions.

Agents represent cells. Basic functions for agent movement and interaction with the environment are implemented in a base class Agent. Derived classes representing specific cells can use these functions, lists of internal variables and parameters and can additionally use custom rules or formulas to define cell dynamics. Agents are updated asynchronously in random order. Hence, changes in one agents' state can influence another agents' dynamics immediately. This reduces numerical errors arising from the discrete time intervals between agents' information exchange and induces stochasticity into the simulation. To reduce errors in integration, especially in the computation of local variables, numerical stability is checked and internal steps are fine grained accordingly. Agents' parameters can be specified to refer to a fixed value for all agents or to be sampled from a random distribution for each agent. Drawing agents' parameters from random distributions induces cellular heterogeneity.

###  Agent Based Model of Sprouting Angiogenesis

The model described here consists of the local variables VEGF-A, sVEGFR1, and sVEGFR1b (sVEGFR1 bound to VEGF-A) and agents representing endothelial cells (ECs). VEGF-A, sVEGFR1, and sVEGFR1b diffuse, are degraded and can associate and dissociate.

We assume the 2D grid considered in simulations to be a subarea of the bottom of a well plate containing culture medium as described in Kappas et al. (2008). We simulate vascular endothelial cells on this grid, originating from two initial cell aggregates representing embryoid bodies. We introduce a VEGF-A influx to each grid cell that depends on the difference between a global reference value (representing the average value in the whole volume of culture medium) and the local value. The reference value is maintained constant as the culture medium in experiments was renewed every 48 h (Kearney and Bautch, 2003).

We model the EC agents according to the intracellular signaling model described in the next section. The EC agents extend or retract *filopodia* based on their active VEGFR2 (*VEGFR*2*a*, see Equations S10, S36, and S37). The *filopodia* determine the radius in which an EC agent senses and binds to VEGF-A. VEGF-A binds to *mVEGFR*1 and *VEGFR*2 according to the respective association and dissociation reactions (Equations S28, S32), *sVEGFR*1 is secreted (Equation S25). An EC cell becomes a tip cell when it satisfies tip cell criteria analogous to criteria described in Blanco et al. (2013):

- *VEGFR*2_*mRNA*>*VEGFR*1_*mRNA*,
- *filopodia*>A^*^,
- $dll4_{mRNA}>D^{*}$

with threshold parameters A^*^ and D^*^. A tip cell determines the direction of the VEGF-A gradient and attempts to move into that direction with a chance of deviating clockwise or counter-clockwise. If no gradient exists, tip cells move into a random direction. The integrity of the sprout is maintained by choosing a random non-tip neighbor when moving. As stalk cells are proliferative, a copy of this neighbor is placed on the tip cells' old position.

###  Intracellular Signaling Dynamics

**Figure 2:**
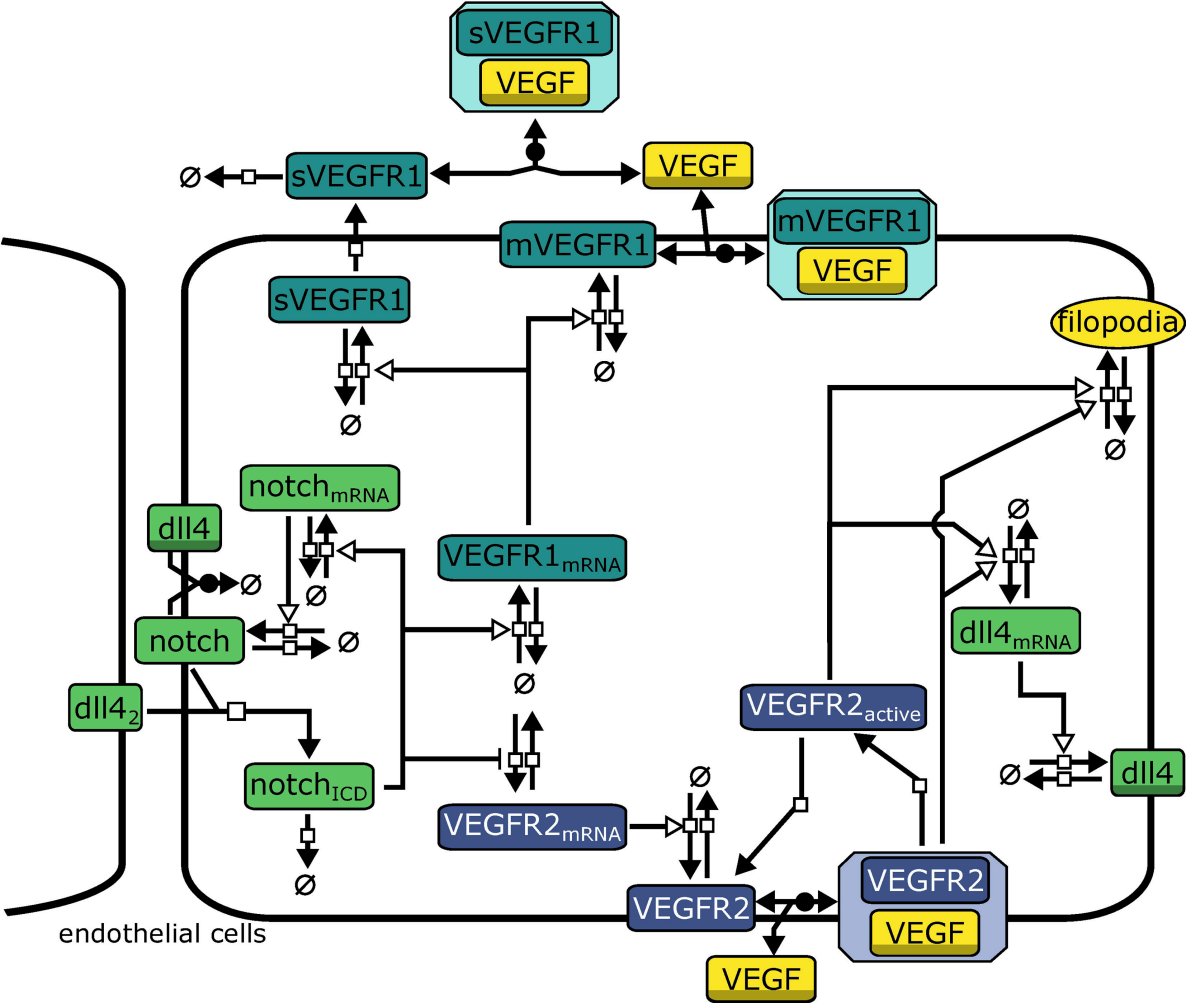
Model topology indicating signaling systems in different colors (green: Dll4-Notch1, turquoise: VEGFR1, purple: VEGFR2). *dll*4_2_ indicates Dll4 from neighboring cells. Symbols adhere to the Systems Biology Graphical Notation format (Novère et al., 2009): filled arrows represent reactions, empty arrowheads represent activation, barred arrows represent inhibition, shaded symbols (dll4, VEGF) indicate the same entity in different processes. Circles crossed by a bar represent sources and sinks of molecule production.

The model describes interactions relevant for tip cell selection and lateral inhibition. It consists of 21 variables and 43 parameters that describe rate laws for binding, transcription, translation, and degradation of molecules, see Figure 2 for an overview. In the agent based model, we use a forward Euler algorithm to solve the corresponding differential equations. The complete model of intracellular interactions and a list of model equations are available as Supplementary Material. The model is provided in the Systems Biology Markup Language (SBML) (Hucka et al., 2003), an XML based format for the exchange of computational models of biological processes. The SBML model has been submitted to the BioModels Database (Chelliah et al., 2015) and assigned the identifier MODEL1804030001.

Binding of *Notch*1 to *Dll*4 from neighboring cells (*Dll*4_*nb*_) releases the active Notch1 intracellular domain *NICD*. *NICD* induces transcription via different downstream factors (Hey2/Hes2). We omit intermediate steps, so *NICD* directly activates transcription of *notch*1_*mRNA* and *VEGFR*1_*mRNA* while repressing expression of *VEGFR*2_*mRNA*. We model transcription rates *v*_*transcription*_ by using Hill-Equations (reviewed, e.g., in Tummler et al., 2014) with additional terms for the activation by an activator *mod*:

(1) $$\begin{array}{r} v_{transcription}=V_{max}\cdot \left(\frac{1}{a}+\left(1-\frac{1}{a}\right)\frac{mod^{h}}{mod^{h}+M_{0}^{h}}\right) \end{array}$$

where *a* indicates the fold increase in transcription upon activation by *mod*, *V*_*max*_ is the maximal transcription rate upon full activation, *M*_0_ determines the modifier value at which *v*_*transcription*_ is half its maximal value and *h* determines the slope of the function. Generally we assume *h* = 2.

All other reactions (translation and degradation, protein binding, and dissociation) follow standard Mass-Action kinetics. VEGFR1 is regarded in two isoforms, membrane-bound VEGFR1 (*mVEGFR*1) and soluble VEGFR1 (*sVEGFR*1). *mVEGFR*1 and *sVEGFR*1 both bind to extracellular VEGF-A, *mVEGFR*1 directly, *sVEGFR*1 only after export. Binding to VEGF-A leads to inactive complexes that can diffuse, degrade, or dissociate. *VEGFR*2 binding to VEGF-A is converted to *VEGFR*2*bound* in the model. After dissociation of VEGF-A, *VEGFR*2 is converted to *VEGFR*2*a*, also representing the activity of the downstream signaling cascade. Both *VEGFR*2*bound* and *VEGFR*2*a* enhance transcription of *dll*4_*mRNA* (omitting intermediate steps in transcriptional regulation). We describe transcription and translation as individual processes for each protein, which also generates time delays.

The model also includes cis-inhibition of Dll4-Notch1 signaling since it has been extensively discussed in literature to be necessary for lateral inhibition (Sprinzak et al., 2010; Shaya and Sprinzak, 2011). Additionally, transcriptional auto-activation of *notch1* (Boareto et al., 2015) has been implied in supporting switch-like behavior by establishing a positive feed-forward loop on the single cell level (Tyson et al., 2003) and we have implemented according reactions in our model (see Equations S2, S16, S17).

###  Model Parameterization

We obtained ranges for parameter values from literature (MacGabhann and Popel, 2006; MacGabhann et al., 2006; Bentley et al., 2008; Hashambhoy et al., 2011; Carlier et al., 2012; Imoukhuede et al., 2013; Boareto et al., 2015; Venkatraman et al., 2016). wherever possible (fold increase in transcription of Dll4 upon VEGFR2 stimulation, the binding affinity of VEGFR1 to VEGF-A relative to the binding affinity of VEGFR2 to VEGF-A, abundance of specific proteins in ECs). Where no specific data was available, we used ranges reported in Schwanhäusser et al. (2011) (ranges for transcription, translation, and degradation rates). In total, 17 initial values have been set, 41 parameters pertaining to intracellular processes have been estimated and 9 parameters pertaining to extracellular processes have been estimated based on 4 data points (relative vessel area and branch points per vessel length for wild type and *VEGFR1 -/-*) from published experimental results (Kappas et al., 2008). Although parameter boundaries were set based on additional literature, we cannot eliminate the possibility that alternative biologically feasible parameterizations could also reproduce the experimental data.

We parameterized the model to reproduce experimental data from Kappas et al. (2008), namely the relative vessel area ($A_{rel}^{EC}$) from mouse embryoid bodies grown in culture wells after 8 days for WT and VEGFR1 -/- mutants. For an overview of published parameter values, values used in this study, and parameter boundaries used, see Table S3 and the notes in the Supplementary SBML model.

To generate the parameterization for the whole model, we first estimated parameters for the intracellular ODE model to generate tip and stalk phenotypes using Copasi (Hoops et al., 2006), then extended the ODE model to describe three cells next to each other and estimated parameters based on the previous iteration to generate lateral inhibition. We used the resulting parameter values as initial guesses for estimating parameters for the whole agent based model. Automatic parameter estimation using a genetic algorithm (Spiesser et al., 2015) was not successful, so we optimized parameter values manually to reproduce experimental data. For this, we iteratively modified individual parameter values and then computed a set of 5 simulations for WT and *VEGFR1 -/-* followed by computing $A_{rel}^{EC}$ and comparison to experimental data. Model fitting was performed forcing all parameters to remain within the range of values reported in literature, which are indicated in Table S3.

For initial values and parameters related to the general condition of a cell (*V*_*max*_ of transcription and translation) and the NICD degradation rate (*k*_*nicd*_*degradation*) we modified not only mean values, but also the deviations of the log-normal distributions from which they are sampled. Parameters related to entity specific characteristics (e.g., binding affinities or fold induction in gene regulation) are not sampled since we assume that the general state of cells is more variable than the entity specific characteristics.

###  Sensitivity Analysis

We analyzed the sensitivity of the simulation results with regard to changes in parameters. We used the percentage of the area occupied by ECs ($A_{rel}^{EC}$) as the model output to analyze. For parameter sensitivity analysis, we consider variations in single parameters only. To analyze the sensitivity of the model output to changes in single parameters, we performed 20 simulations per parameter value over a range of parameter values centered around the value originally used. This has been done for 46 parameter values between 75% and 125% of the original value of each parameter (except for the exponents in the Hill equations). Results are available as Figures S2–S47. For specific parameters, we repeated the analysis using 51 different parameter values from 1 to 199% of the original value (depicted in **Figures 4**, **5**). This analysis depends on the parameterization chosen and the effects of changes in two (or more) parameters can not be predicted from combining the effects of two individual parameter changes.

### Branching Analysis

To measure branch points, we saved simulation results as image files and analyzed these with ImageJ-MATLAB (Hiner et al., 2017). Images were smoothened to ensure connectivity of agents, then binarized and skeletonized. The skeletonized path was analyzed for branch points using AnalzyeSkeleton (Arganda-Carreras et al., 2010). Branch points per millimeter vessel length were computed using the total length of all branches from the ImageJ analysis.

## Results

### *In-silico* Model Reproduces Experimental Data on Wild Type and VEGFR1 -/- Strain

**Figure 3:**
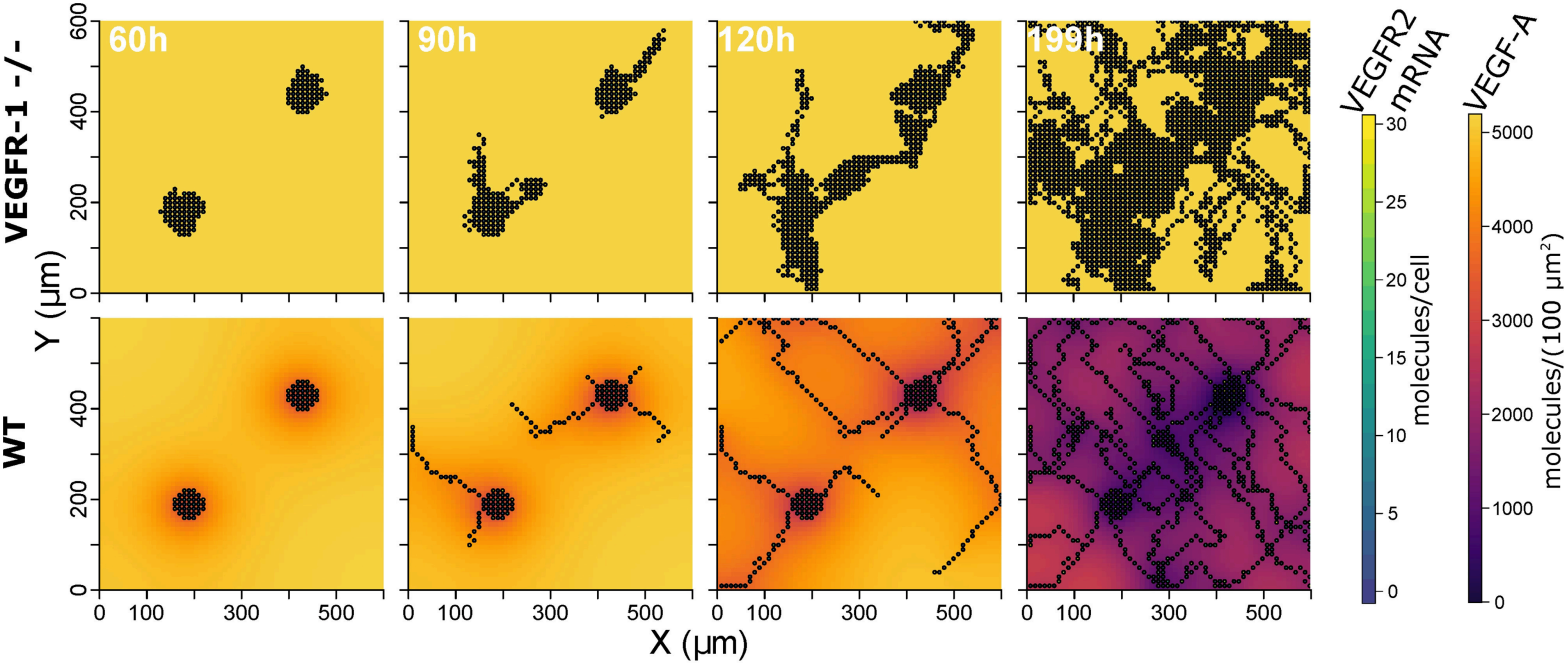
Representative simulation results: extracellular VEGF-A, and VEGFR2 mRNA for VEGFR1 -/- strain **(top)** and wild type **(bottom)** at 60, 90, 120, and 199 h (from left to right).

The constructed model with the described parameterization reproduces experimental data on mouse embryoid bodies of wild type and *VEGFR1*-/- strains as described in Kappas et al. (2008). Over 20 simulation runs for wild type conditions, we observed distinct vessels in a network topology and ECs covered 17.2% ± 5.8 (mean ± standard deviation) of the simulated area, compared to 16.7% reported for experiments. In 20 simulations for *VEGFR1*-/- conditions, nearly no distinct structures were apparent and ECs covered 55.1% ± 10.5 area (55.34% in experiments). *Branch points*/*mm* are also comparable to experimental data (9.9 ± 2.2 *branchpoints*/*mm* predicted, 12.6 ± 3 *branchpoints*/*mm*). Figure 3 shows representative simulation results for the wild type and the *VEGFR1*-/- strain, comparable to the experimental results shown in Kappas et al. Kappas et al. (2008). Movies of the simulations are given in Supplementary Material.

Initial endothelial cells in the model are seeded at two locations on the grid, corresponding to the seeding of multiple embryoid bodies (Kappas et al., 2008). Simulations with one initial cell group in the center of the grid yielded similar means for the relative area covered by ECs ($A_{rel}^{EC}$) in the WT and *VEGFR1*-/- strain but substantially wider standard deviations (see Supplementary Material for details and model files).

The transition between tip and stalk cells is smooth. Although the mean values of intracellular variables strongly differ for tip and stalk cells, they overlap: non-tip cells exist for which some intracellular variables have similar values as generally observed in tip cells. Exemplary distributions are given in Figure S1.

###  Contributions of VEGF-A Receptors

To analyze the contribution of the different VEGF-A receptors to sprouting, we evaluated the sensitivity of $A_{rel}^{EC}$ to changes in transcription and translation rates of the receptors. The parameters analyzed are the rate parameter of translation and the *V*_*max*_ parameter of Equation (1) for transcription.

**Figure 4:**
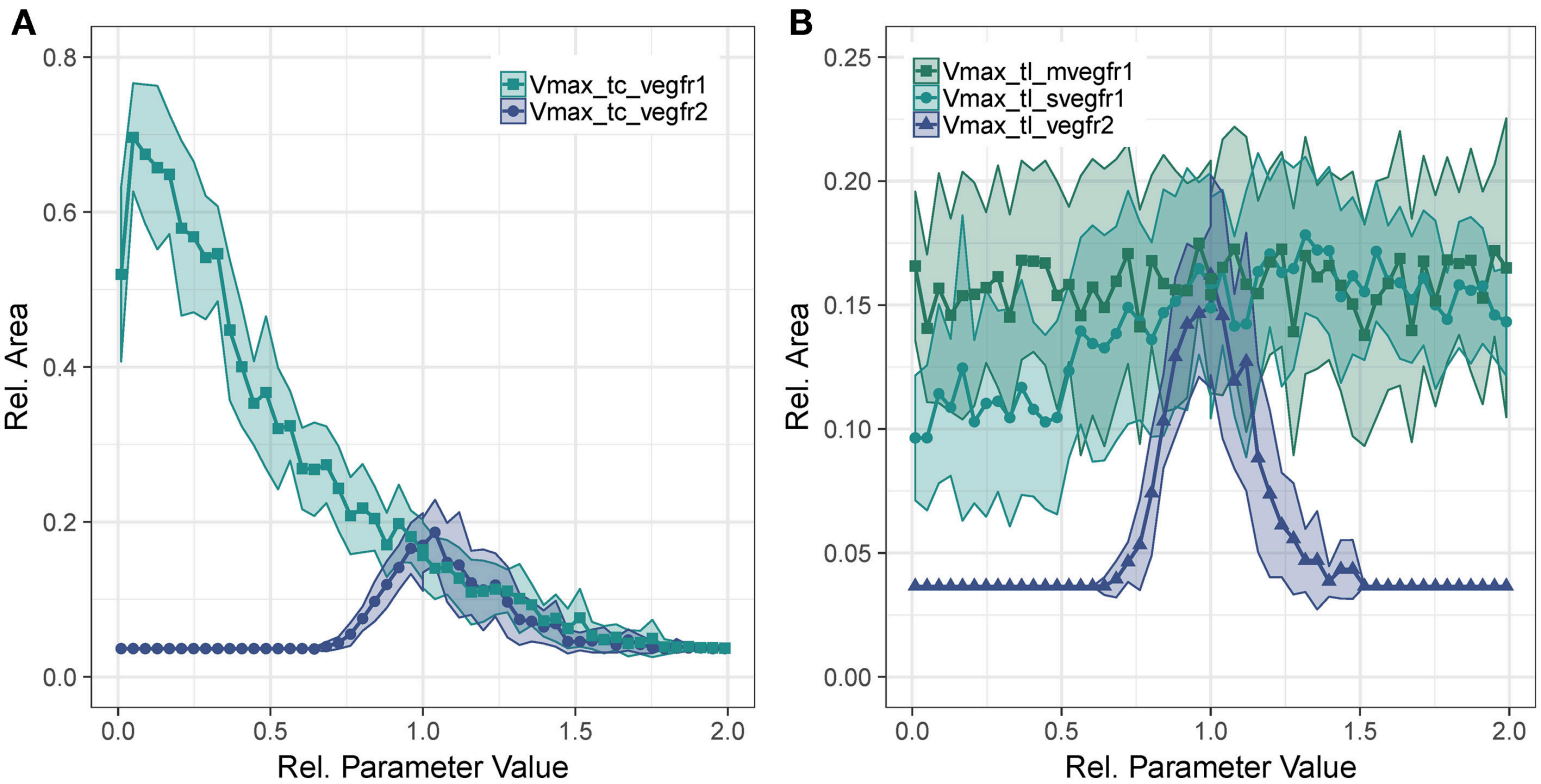
Parameter sensitivity: changes in relative vessel area ($A_{rel}^{EC}$) after 192 h for variation in parameter *V*_*max*_ for *VEGFR2* and *VEGFR1* transcription **(A)**, and the translation rate of mVEGFR1, sVEGFR1, VEGFR2 **(B)**.

Concerning *V*_*max*_ of transcription for VEGFR2, we observe that too little VEGFR2 transcription renders cells insensitive to VEGF-A while too much VEGFR2 leads to a hyperactivation of Dll4 and thus too high NICD levels that suppress sprouting. This is true for both VEGFR2 transcription (Figure 4A) as well as for VEGFR2 translation (Figure 4B). As shown in Figure 4A, the sensitivity of $A_{rel}^{EC}$ to VEGFR1 transcription displays a strong inverse relationship. Increased VEGFR1 transcription causes a reduction in tip cell phenotypes (a tip cell needs to satisfy *VEGFR*2_*mRNA*>*VEGFR*1_*mRNA*) and a reduction in available VEGF-A through binding of VEGF-A to VEGFR1 isoforms. For a complete knockout of VEGFR1, the $A_{rel}^{EC}$ decreases from 69 to 51%. This is due to the directional cues that VEGFR1 provides. Under complete VEGFR1 knockout, vessels grow in random directions and cannot fill space as effectively as if they would grow more orthogonally.

Figure 4B shows the effect of changes in the rate of translation of VEGF receptors. The effect for VEGFR2 is comparable to that of changing transcription rate (compare Figure 4A), only that the range of parameter values that allows for sprouting is narrower. An effect of changing the translation of mVEGFR1, the membrane bound form of VEGFR1, is not detectable. There is a weak positive correlation between the translation of sVEGFR1 and the relative vessel area. The sensitivity of *branch points*/*mm* exhibits similar trends as the sensitivity of the relative vessel area (Figures S49–S52).

###  Lateral Inhibition via Dll4-Notch1 Signaling

**Figure 5:**
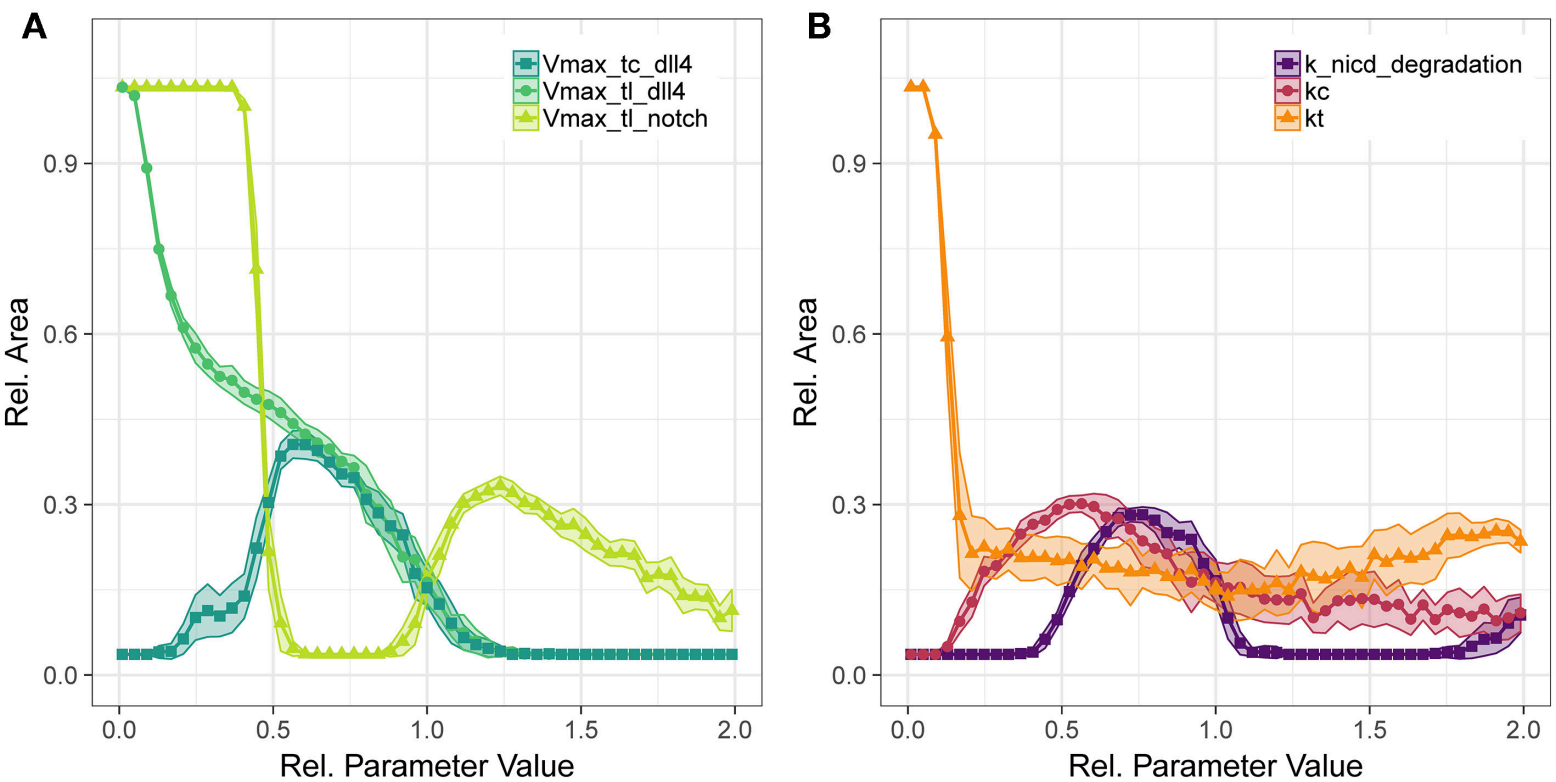
Parameter sensitivity: changes in relative vessel area ($A_{rel}^{EC}$) after 192 h for variation in the translation rates of Notch1 and Dll4, and the transcription rate of Dll4 **(A)**, and the degradation rate of notch1 intracellular domain and the parameters for Dll4-Notch1 association in *cis* (kc) and *trans* (kt) **(B)**.

We have also analyzed the translation parameters of Dll4 and Notch1 (Figure 5A). The translation of Dll4 is anti-correlated to $A_{rel}^{EC}$ since an increase in Dll4 leads to an increase in *trans* activation of Notch1 which in term suppresses VEGFR2 transcription and thus prevents sprouting. The sensitivity to changes in the transcription rate of *dll4* (Figure 5A) does not resemble the sensitivity for the translation rate. This is due to the tip cell criterion $dll4_{mRNA}>D^{*}$, *dll4* mRNA has to exceed a threshold for sprouting. Accordingly, there is a positive correlation between $A_{rel}^{EC}$ and *dll4* transcriptional rate until the *trans* activation of Notch1 outweighs the tip cell activating influence of *dll4*, at around half the original parameter value. For higher values of *dll4* transcription, the sensitivity resembles that of Dll4 translation. The same trends hold for *branch points*/*mm* (Figures S51),

The translation of Notch1 shows a different pattern: For *V*_*max*_ of Notch1 translation below half the original value, no *trans* activation occurs and hence no inhibition of VEGFR2 occurs and vessels fill the complete area. Between half the original parameter value to almost the original value, lateral inhibition is sufficient to lead to VEGFR1 expression, but not sufficient to induce patterning and sprouting. The increase in relative area beyond the original value indicates functional lateral inhibition and patterning that reach a maximum of about 30% $A_{rel}^{EC}$. Further increase of Notch1 production makes it more unlikely to overcome tip cell inhibition, so that the relative EC area decreases. The sensitivity to transcriptional regulation of *notch1* is similar to that of translational regulation (see Figures S40, S45), because *notch1* mRNA is not involved in tip cell phenotype conditions, as are *VEGFR1, VEGFR2*, and *dll4* mRNA. Again, the *branch points*/*mm* over the parameter ranges follow the trends in relative vessel area (Figure S51).

Concerning the interactions in Notch1-Dll4 signaling, we have analyzed the sensitivity to parameters kc, kt, and the degradation rate of NICD, depicted in Figure 5B. Parameter kc resembles the binding rate of Dll4 to Notch1 on the same cell, leading to *cis* inhibition of Notch1. Parameter kt describes the binding rate of Dll4 to Notch1 on a neighboring cell, leading to *trans* activation of Notch1. The *trans* activation determined through kt is necessary for lateral inhibition and its absence leads to hypersprouting. Conversely, a value of kc above 0.154 is necessary to reduce lateral inhibition and induce sprouting.

The amount of NICD, determined by Notch1 activation and NICD degradation, is crucial for balancing VEGFR1/VEGFR2 levels and thus determine the degree of lateral inhibition (as indicated in Figure 2). As shown in Figure 5, NICD degradation needs to surpass a threshold (0.385 of the estimated value) to allow sufficient production of VEGFR2 and thus allow sprouting at all. If NICD degradation exceeds another threshold (1.192 of the estimated value), *VEGFR2* transcription is not reduced sufficiently, lateral inhibition ceases and no tip cells are formed. For values of NICD degradation above 1.692 times the estimated value, the transcriptional activation of *VEGFR1* is sufficiently reduced to induce sprouting again, but in the absence of lateral inhibition.

For the Notch1-Dll4 signaling interactions, parameter variations lead similar trends for *branch points*/*mm* as for the relative vessel area (compare Figure S52).

**Figure 6:**
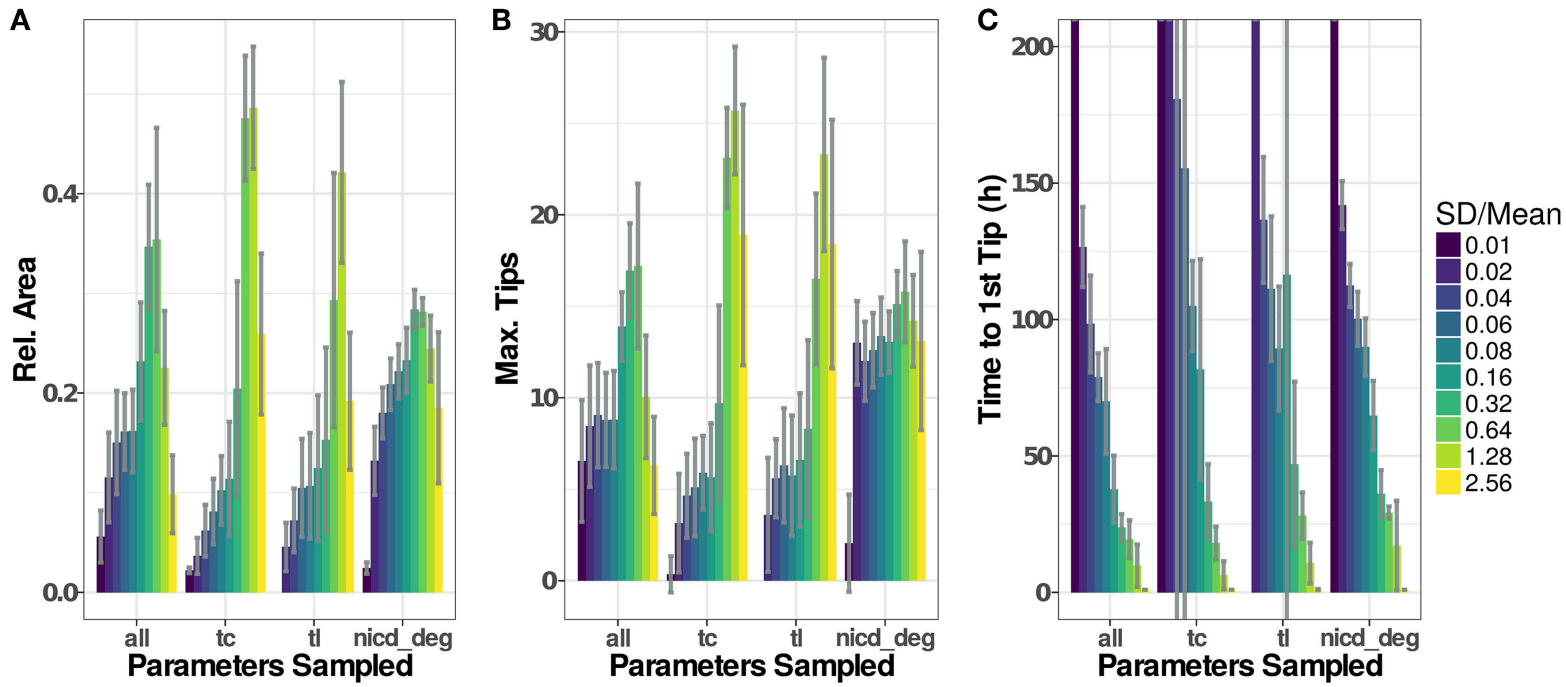
Parameter distributions strongly influence model dynamics. The standard deviation of the parameter distributions were varied from 0.01 to 2.56·μ. Sampled parameters are: *V*_*max*_ of transcription (tc), translation rates (tl), and the degradation rate of NICD (nicd_deg) or all of the above. Initial values for new agents were sampled accordingly under all scenarios. Model output for 20 simulations of each setting have been recorded as $A_{rel}^{EC}$ **(A)**, the maximal number of active tip cells **(B)**, and the time of the first tip cell **(C)**. If no tip cells occur during a simulation run (199 h), we set the time to the first tip cell to 1,000 h. Plotted values are given in Table S2.

For most parameters in the Dll4-Notch1 signaling system, the maximal value of $A_{rel}^{EC}$ is about 30%, because changes in Dll4-Notch1 signaling affect the timing of the onset of sprouting in our simulations, not the overall number of sprouts, which is more strongly driven by the availability of active VEGFR2 (compare also Figures 6B,C).

###  Influence of Cell-to-Cell Variability

To assess the influence that the sampling of parameters has on simulation results, we have analyzed simulation runs using distributions with similar means but different standard deviations, as depicted in Figure 6. Generally, low variability leads to low $A_{rel}^{EC}$ and late emergence of tip cell phenotypes, often without any tip cells during the simulated time course. Here, the high homogeneity of the cells and the symmetric initial conditions impose highly similar conditions on each agent so that emergence of tip cells depends solely on the stochasticity induced through the asynchronous agent updates. Increasing the width of the random distributions for transcriptional and translational parameters leads to an increase in sprouting up to a ratio $\frac{SD}{Mean}=0.64$, beyond which both Dll4-Notch1 and VEGF-A signaling do not function properly anymore and tip cell phenotypes emerge randomly. This leads to an almost instantaneous emergence of the first tip cell for $\frac{SD}{Mean}>1$ (Figure 6C) and $A_{rel}^{EC}$ at or below the maximal value reached (Figure 6A).

For the degradation of NICD, an increased variability leads to a quite stable $A_{rel}^{EC}$ as well as a stable maximal number of tips. This might seem surprising because the previous analysis showed that sprouting only occurs in a very tight range of values for the NICD degradation rate, as shown in Figure 5B. The results of the sensitivity analysis however impose the same parameter value on all cells in the model while the variability analyzed here is between cells. The degradation rate of NICD critically determines a cells chance of becoming a tip cell, especially if values of neighboring cells differ and thereby reinforce lateral inhibition.

The variability of the model output does not increase additively when sampling transcriptional rate parameters, translational rate parameters and the NICD degradation rate (“all” in Figure 6). Instead, the model output becomes more stable against increasing parameter variability compared to the “tc” and “tl” groups for intermediate ratios of $\frac{SD}{Mean}$, because pattern formation is reinforced by the stronger lateral inhibition caused by the variability in the NICD degradation rate. For higher rates of $\frac{SD}{Mean}$, the signaling fidelity breaks down and tip cells arise almost randomly as in the “tc” and “tl” groups, slightly stabilized by the influence of the variability in NICD degradation that has a stable influence over all ratios of $\frac{SD}{Mean}$.

Again, the number of branch points by vessel length follows the trends observed in the $A_{rel}^{EC}$.

## Discussion

Angiogenesis is a multi-scale process where multiple signals must be integrated to enable cellular decision making leading to the sprouting of new vessels. Here, we present an agent based computational model of sprouting angiogenesis that focuses on intracellular signaling. We show that the release of the decoy receptor sVEGFR1 has two distinct effects. By reducing VEGF-A concentrations in the vicinity of stalk cells, VEGFR1 (1) reduces sprouting and (2) directs sprouts to grow orthogonal to existing structures. The second effect leads to a faster vascularization of an avascular area under homogeneous VEGF-A conditions. We observe this impact of VEGFR1 under a wide range of parameters. Our results are consistent with previous findings on the interaction of VEGF and sVEGFR1 in a static environment (Hashambhoy et al., 2011; Chappell et al., 2016), but our study extends on this by considering a dynamic environment in which production rates as well as cell positions are not fixed but emerge from model dynamics.

Our analysis also reveals that the system dynamics, especially the timing of sprouting, strongly depend on the parameterization of the Dll4-Notch1 signaling system that determines lateral inhibition. Our model predicts that the stability of the notch intracellular domain strongly influences the timing of sprouting without a strong impact on vessel network architecture.

We furthermore show that cellular heterogeneity is an important driver of pattern formation. The case of NICD in our model highlights that varying cell-to-cell variation of a parameter has different effects than varying the magnitude of the same parameter. This also has implications for experimental research, because it is accordingly not sufficient to know the mean value of some parameter in an ensemble of multiple cells but we also need to know its variability specified by the distribution and its moments.

###  Limitations of the Approach

The model presented here is a 2D lattice based model with fixed cell shapes. This means that its predictions are limited to 2D environments and cannot be directly transferred to clinical conditions, e.g., in tumor growth.

Finding the appropriate balance of model detail or resolution is a fundamental challenge in modeling. Any increase in model complexity increases the amount of experimental data necessary to find a reliable parameterization. Here, we have not included all molecules involved in VEGF signaling in sprouting angiogenesis (see e.g., Ferrara et al., 2003; Cao, 2009 for reviews of involved mechanisms), but have attempted to limit our model to the most important mechanisms with respect to the interplay between VEGF, VEGFR1 and VEGFR2. Nevertheless, the model contains 58 parameters, some of which are not fixed values but sampled from random distributions. For none of the parameters, we could find a reliable value in literature that is certainly applicable to the experimental conditions the model describes. Rather, we use the available experimental data (relative vessel area and branch points per vessel length for WT and *VEGFR1 -/-*) to generate a parameterization that is within sensible boundaries based on literature data.

This parameterization is not guaranteed to be equal to the physiological parameterization. Because we have restricted parameter boundaries based on experimental data and our data reproduces findings reported in literature (Hashambhoy et al., 2011; Chappell et al., 2016), we expect the model analysis to yield at least qualitatively correct predictions.

###  VEGFR1 in Sprouting Angiogenesis

Kearney et al. (2002) have shown an increase of relative EC area and a loss of vascular network properties in *VEGFR1*-/- mutants in *in-vitro* cultures of mouse embryonic bodies. These defects can be restored by additional expression of sVEGFR1 (Roberts et al., 2004), but not mVEGFR1 Kappas et al. (2008), and are VEGF-dependent (Chappell et al., 2009). The proposed mechanism of action is that sVEGFR1 generates a tight corridor of VEGF-A to guide the tip cell away from stalk cells.

The analysis of our model indicates that VEGFR1 suppresses sprouting by reducing the global VEGF-A concentration with increasing cell density (compare the upper and lower row of simulation results in Figure 3) and that VEGFR1 provides spatial cues by reducing the local VEGF-A concentration around stalk cells, which improves vessel network topology. These spatial cues lead to nearly orthogonal sprout growth instead of random sprout growth that we observe when VEGFR1 transcription is removed completely. This also explains the decrease in relative vessel area when removing VEGFR1 transcription completely in Figure 4A. These spatial cues are also the reason for the weak positive correlation between sVEGFR1 translation and relative vessel area in Figure 4B, indicating that sVEGFR1 has a stronger effect than mVEGFR1. Although our model implies that these effects contribute under a wide range of parameters, the importance of either of these mechanisms depends on the external conditions. Under wound healing conditions with cellular sources of soluble VEGF-A, the importance of VEGFR1 will be higher than in the developing mouse retina, where new vessels follow the tracks of astrocytes supplemented with ECM-bound VEGF-A (Gerhardt et al., 2003).

Diffusion properties and morphogen gradients are notoriously difficult to quantify in tissues experimentally (Bothma et al., 2010; Köhn-Luque et al., 2011). Our computational analysis of the role of sVEGFR1 provides important insights into the regulatory capabilities of this mechanism and opens up new questions: In contrast to the static environment considered in our simulations, how does the dynamic environment provided by the ECM influence the effects of VEGFR1? This question is of specific importance to clinical settings, e.g., regeneration or tumor growth, where multiple dynamic sources of VEGF-A might be present and the ECM is undergoing active rearrangement.

Also, our analysis indicates that only the complete removal of VEGFR1 transcription abolishes the spatial cues provided by sVEGFR1 (Figure 4A). This is an artifact of our model, because our *in-silico* cells sense any difference in external VEGF concentrations, unlike *in-vitro* or *in-vivo* situations, where cells sense the VEGF-A gradient by differential binding of VEGF-A to receptors on their filopodia.

###  Lateral Inhibition and Cellular Heterogeneity

In contrast to the gradual changes in simulation output caused by changes in VEGFR1 or VEGFR2 production rates, changes in parameters involved in Delta-Notch1 signaling cause stronger and more abrupt changes over certain parameter value ranges (Figures 4, 5). This indicates that the fine tuning of parameters in lateral inhibition is more important in angiogenesis than the actual VEGF-A receptor kinetics, according to our analysis.

In the model we present, the base state of a cell is a quiescent stalk-cell like state, and the basic activity of lateral inhibition is relatively high. For sprouting, cells have to overcome this basal lateral inhibition. Cellular heterogeneity through sampled parameters is an important driver of the differentiation of individual cells in our model. Although the sampling is random, a specific cell can be primed for sprouting by the sampled parameter values. Neighboring cells might be assigned parameter values that make them less likely to sprout and the difference between these cells is then further amplified through lateral inhibition. The most important parameter to generate heterogeneity for sprouting is the degradation rate of NICD. Even small stochasticity in NICD degradation increases cellular heterogeneity in the lateral inhibition pathway and significantly improves sprouting efficiency. Because the stability of NICD does not directly affect sprouting but the heterogeneity between neighboring cells, larger stochasticity in NICD degradation does not lead to a breakdown of the system, as observed when sampling transcription or translation parameters from wide distributions (Figure 6). The importance of the regulation of NICD stability for signaling has been discussed before (see e.g., Kopan and Ilagan, 2009; Andersson et al., 2011; Herbert and Stainier, 2011; Bray, 2016), mechanisms include different cleavage products and active targeting of NICD for proteosomal degradation, acetylation by SIRT1 or interaction with other signaling pathways via YAP or SMAD3.

Beyond this specific finding, our analysis on the role of parameter distributions in computational models of multicellular ensembles also has a general relevance. Assuming a fixed value for NICD degradation for all cells and varying this value showed only a narrow band of parameter values that lead to efficient sprouting (Figure 5B). Sampling the value of NICD degradation for each cell from a random distribution improves sprouting efficiency (Figures 6A,B). Our simulations show that pattern formation is facilitated by cell-cell heterogeneity. It is not surprising that this effect is most pronounced for a parameter at the core of lateral inhibition, the main driver of pattern formation under the conditions used here. Generally, pattern formation can be reinforced or damped by cellular heterogeneity and this heterogeneity needs to be considered, in computational and experimental studies alike. Beyond transcriptional noise and asymmetric cell division (Costa et al., 2016), we need to consider that cell-cell and cell-matrix interactions also modify and influence cellular signaling (Qazi et al., 2017) and even the overall cellular state (Battich et al., 2015).

###  Computational Modeling of Sprouting Angiogenesis

The agent based model we present here omits cell shape dynamics and ensures vessel connectivity by dividing stalk cells upon tip movement. This neglects cell shape changes and their effect on network formation (Boas and Merks, 2015). Nevertheless, the model presented here considers biochemical signaling with a higher level of detail than most models of sprouting angiogenesis (Merks et al., 2004; Bentley et al., 2008, 2009; Checa and Prendergast, 2010; Jakobsson et al., 2010; Carlier et al., 2012, 2014; Köhn-Luque et al., 2013; van Oers et al., 2014; Boas and Merks, 2015; Walpole et al., 2015; Ubezio et al., 2016; Venkatraman et al., 2016; Bentley and Chakravartula, 2017). The introduction of separate transcription/translation steps in our model—although it still omits intermediate steps in transcriptional regulation—introduces a dynamic delay between transcriptional activation and protein production that is omitted in most models. Because of the more faithful biochemical model, experimental perturbations can be more directly linked to model entities. The coarse spatial resolution of our model can be interpreted as a different level of magnification from which the biological dynamics are observed.

The complete model code can be downloaded and reused and we hope that further development of the model by us and other groups will help to further understand the dynamics of sprouting angiogenesis. Future development of the model could focus on the interactions between chemical and mechanical signals or in the cross-talk between different signaling pathways.

In order to generate reliable and clinically relevant predictions of angiogenesis to improve regeneration, enable fine-tuned tissue engineering or ablate tumor-dependent angiogenesis, we need to integrate various mechanisms in a dynamic environment. Moreover, these models need to be parameterized based on dedicated experimental data. By providing our model in an open and reproducible manner, we enable its integration into models that consider cell shape dynamics, interactions with the ECM, or network formation in 3D.

###  Conclusion and Outlook

We present an agent based computer model of sprouting angiogenesis focusing on intracellular signaling and its effects on pattern formation. Specifically, we focus on the role of VEGFR1 and its interrelation with VEGFR2 and Delta-Notch signaling. Our findings do not replace published modeling efforts but are complementary because our focus differs from existing literature. Our simulations go beyond comparable models concerning level of detail of intracellular signaling, cell numbers considered, and simulation time.

Our results support the hypothesis that soluble VEGFR1 provides spatial cues to guide sprouts away from their origin and it predicts that this effect is robust over a wide range of parameters. Our model analysis shows that lateral inhibition, and especially the regulation of NICD stability and its variability across cells, is critical for the regulation of sprouting angiogenesis.

This leads us to argue that complementary modeling approaches need to combined and supplemented with dedicated experimental data to generate reliable predictions, to generate models that do not only reproduce training data but are also capable of predicting validation data correctly and function over a wide range of conditions. The advantage of incorporating dedicated signaling models into cell-based models of angiogenesis is that these models provide direct links between experimental perturbations and model dynamics.

## Data Availability

Source Code for the simulator and analysis can be found at https://gitlab.com/ModularABM/AngioABM/tree/VEGFR1 and https://gitlab.com/ModularABM/ABMTools/tree/VEGFR1. The model of intracellular dynamics has been submitted in SBML format to the biomodels repository (Chelliah et al., 2015) and assigned the identifier MODEL1804030001. All other data for this study is included in the manuscript and Supplementary Material.

## Author Contributions

CK and SC designed the study, wrote and revised the manuscript, read the manuscript, and approved its content. CK implemented simulator and analysis software, constructed and parameterized models, and analyzed simulation results.

### Conflict of Interest Statement

The authors declare that the research was conducted in the absence of any commercial or financial relationships that could be construed as a potential conflict of interest.
