# Supplementary material for: Computational Modeling to Quantify the Contributions of VEGFR1, VEGFR2, and Lateral Inhibition in Sprouting Angiogenesis
Source: Front Physiol. 2019 Mar 27;10:288. doi: 10.3389/fphys.2019.00288 (PMC6445957; doi:10.3389/fphys.2019.00288)
Supplement: Supplementary file 12 [file Data_Sheet_1.PDF]

Supplementary Material: Computational modeling to quantify the contributions of VEGFR1, VEGFR2, and lateral inhibition in sprouting angiogenesis

1 DISTRIBUTIONS OF INTRACELLULAR VARIABLES FOR TIP AND STALK CELLS

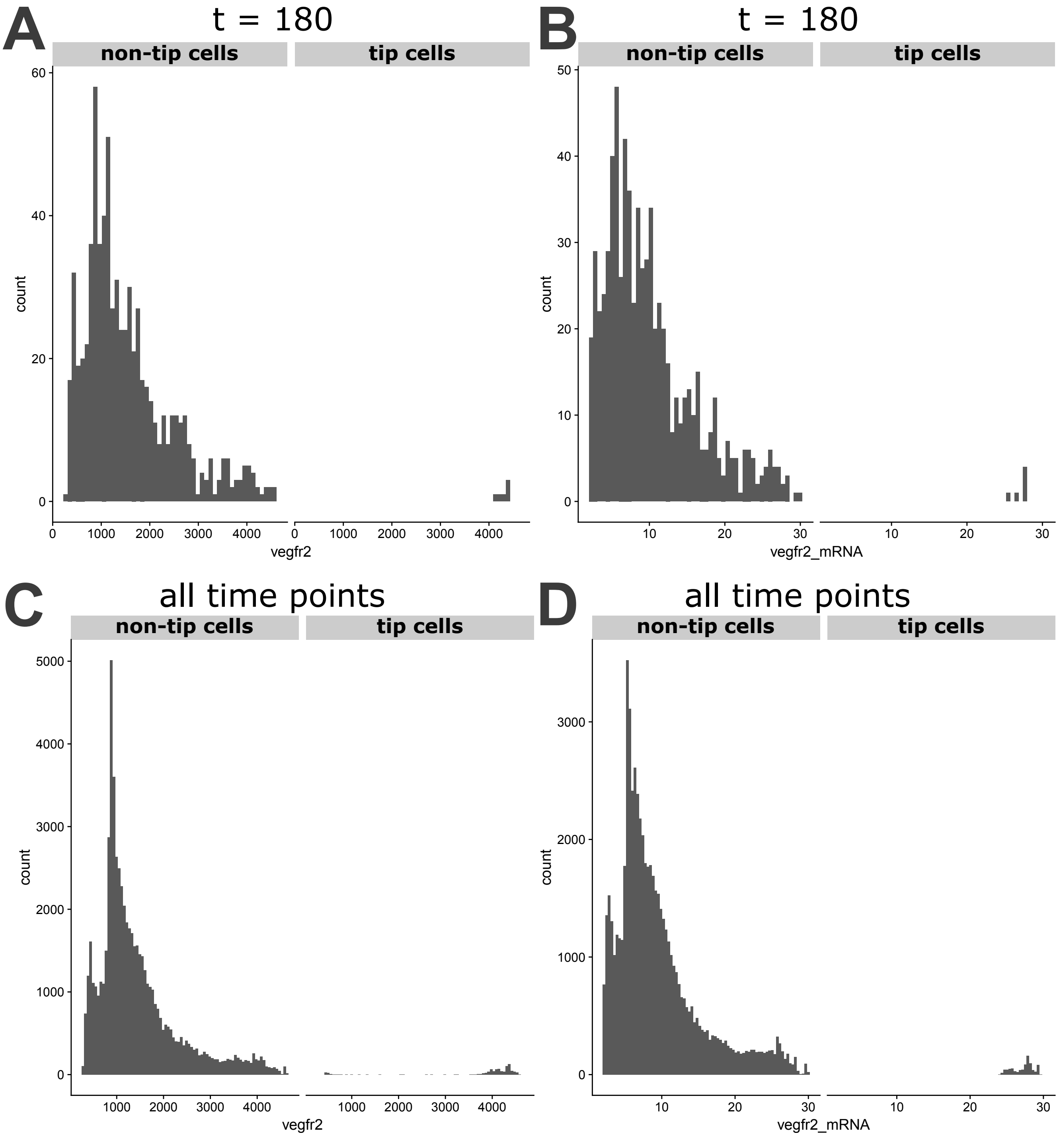

Figure S1: Histograms of intracellular VEGFR2 (A,C) and VEGFR2mRNA (B,D) for the wild type simulation (as used for generating Figure 3 in the main text), either at simulation time t = 180 (A,B) or over all time points (C,D), differentiated for tip and non-tip cells. Although the means of the distributions for tip and stalk cells are well separated, there are stalk cells with similar vegfr2 and vegfr2_mRNA numbers.

2 LIST OF MOVIES

Supplemental Material contains a number of movies showing simulation results over time. We have used the same simulations as used in the preparation of Figure 3 of the main text for the movies. The movies have been tested to run with VLC 3.0.2.

All movies are named like `VEGFR1_movie_STRAIN_ENTITY`, where `STRAIN` is either WT (wild type) or KO (*VEGFR1 -/-*) and `ENTITY` indicates the model entity plotted. The following movies are contained in the Supplemental Material:

- **VEGFR1_movie_WT_dll4_mRNA.mp4**
- **VEGFR1_movie_WT_nicd.mp4**
- **VEGFR1_movie_WT_vegfr1_mRNA.mp4**
- **VEGFR1_movie_WT_vegfr2_mRNA.mp4**
- **VEGFR1_movie_KO_dll4_mRNA.mp4**
- **VEGFR1_movie_KO_nicd.mp4**
- **VEGFR1_movie_KO_vegfr1_mRNA.mp4**
- **VEGFR1_movie_KO_vegfr2_mRNA.mp4**

3 EQUATIONS OF EC AGENTS' INTERNAL DYNAMICS

3.1 Differential Equations

Differential equations used to compute the changes in agents' internal variables. Internal variables are in molecules per cell. Parameter values, units, and additional information can be found in Supplemental Tables S1, S2, and S3.

$$\frac{\mathrm{d}dll4}{\mathrm{d}t} = -v^{D/N}_{cisInihibition} + v^{Dll4}_{translation} - v^{Dll4}_{degradation} \tag{S1}$$

$$\frac{\mathrm{d}notch}{\mathrm{d}t} = -v^{D/N}_{cisInihibition} + v^{notch}_{translation} - v^{notch}_{degradation} - v^{D/N}_{transActivation} \tag{S2}$$

$$\frac{\mathrm{d}NICD}{\mathrm{d}t} = v^{D/N}_{transActivation} - v^{NICD}_{degradation} \tag{S3}$$

$$\frac{\mathrm{d}sVEGFR1}{\mathrm{d}t} = v^{sVEGFR1}_{translation} - v^{sVEGFR1}_{degradation} - v^{sVEGFR1}_{secretion} \tag{S4}$$

$$\frac{\mathrm{d}mVEGFR1}{\mathrm{d}t} = v^{mVEGFR1}_{translation} - v^{mVEGFR1}_{degradation} - v^{mVEGFR1}_{vegf\_binding} + v^{mVEGFR1b}_{dissoc} \tag{S5}$$

$$\frac{\mathrm{d}mVEGFR1b}{\mathrm{d}t} = v^{mVEGFR1}_{vegf\_binding} - v^{mVEGFR1b}_{dissoc} \tag{S6}$$

$$\frac{\mathrm{d}VEGFR2}{\mathrm{d}t} = v^{VEGFR2}_{translation} - v^{VEGFR2}_{degradation} - v^{VEGFR2}_{vegf\_binding} + v^{VEGFR2a}_{inactivation} \tag{S7}$$

$$\frac{\mathrm{d}VEGFR2b}{\mathrm{d}t} = v^{VEGFR2}_{vegf\_binding} - v^{VEGFR2b}_{dissoc} \tag{S8}$$

$$\frac{\mathrm{d}VEGFR2a}{\mathrm{d}t} = v^{VEGFR2b}_{dissoc} - v^{VEGFR2a}_{degradation} - v^{VEGFR2a}_{inactivation} \tag{S9}$$

$$\frac{\mathrm{d}filopodia}{\mathrm{d}t} = v^{filopodia}_{extension} - v^{filopodia}_{activeRetraction} - v^{filopodia}_{basalRetraction} \tag{S10}$$

$$\frac{\mathrm{d}dll4\_mRNA}{\mathrm{d}t} = v^{dll4\_mRNA}_{transcription} - v^{dll4\_mRNA}_{degradation} \tag{S11}$$

$$\frac{\mathrm{d}notch\_mRNA}{\mathrm{d}t} = v^{notch\_mRNA}_{transcription} - v^{notch\_mRNA}_{degradation} \tag{S12}$$

$$\frac{\mathrm{d}VEGFR1\_mRNA}{\mathrm{d}t} = v^{VEGFR1\_mRNA}_{transcription} - v^{VEGFR1\_mRNA}_{degradation} \tag{S13}$$

$$\frac{\mathrm{d}VEGFR2\_mRNA}{\mathrm{d}t} = v^{VEGFR2\_mRNA}_{transcription} - v^{VEGFR2\_mRNA}_{degradation} \tag{S14}$$

3.2 Reaction-Diffusion Equations

Reaction-diffusion equations used in the differential equations in 3.1.

$$v^{D/N}_{cisInihibition} = k_c \cdot Notch \cdot Dll4 \tag{S15}$$

$$v^{D/N}_{transActivation} = k_t \cdot Notch \cdot Dll4_{nb} \tag{S16}$$

$$v^{NICD}_{degradation} = k_{-NICD} \cdot NICD \tag{S17}$$

$$v^{Dll4}_{translation} = k_{+Dll4} \cdot dll4\_mRNA \tag{S18}$$

$$v^{Dll4}_{degradation} = k_{-Dll4} \cdot Dll4 \tag{S19}$$

$$v^{notch}_{translation} = k_{+Notch} \cdot notch\_mRNA \tag{S20}$$

$$v^{notch}_{degradation} = k_{-Notch} \cdot Notch \tag{S21}$$

$$v^{sVEGFR1}_{translation} = k_{+sVEGFR1} \cdot VEGFR1\_mRNA \tag{S22}$$

$$v^{sVEGFR1}_{degradation} = k_{-sVEGFR1} \cdot sVEGFR1 \tag{S23}$$

$$v^{sVEGFR1}_{secretion} = k_{sVEGFR1,export} \cdot sVEGFR1 \tag{S24}$$

$$v^{mVEGFR1}_{translation} = k_{+mVEGFR1} \cdot VEGFR1\_mRNA \tag{S25}$$

$$v^{mVEGFR1}_{degradation} = k_{-mVEGFR1} \cdot mVEGFR1 \tag{S26}$$

$$v^{mVEGFR1}_{vegf\_binding} = k_{mR1:V} \cdot mVEGFR1 \cdot VEGF_{here} \tag{S27}$$

$$v^{mVEGFR1b}_{dissoc} = k_{R-V} \cdot mVEGFR1b \tag{S28}$$

$$v^{VEGFR2}_{translation} = k_{+VEGFR2} \cdot VEGFR2\_mRNA \tag{S29}$$

$$v^{VEGFR2}_{degradation} = k_{-VEGFR2} \cdot VEGFR2 \tag{S30}$$

$$v^{VEGFR2}_{vegf\_binding} = k_{mR2:V} \cdot VEGFR2 \cdot VEGF_{here} \tag{S31}$$

$$v^{VEGFR2b}_{dissoc} = k_{R-V} \cdot VEGFR2b \tag{S32}$$

$$v^{VEGFR2a}_{inactivation} = k_{\sim VEGFR2a} VEGFR2a \tag{S33}$$

$$v^{VEGFR2a}_{degradation} = k_{-VEGFR2a} VEGFR2a \tag{S34}$$

$$v^{filopodia}_{extension} = k_{f,1} \cdot VEGFR2aa \tag{S35}$$

$$v^{filopodia}_{activeRetraction} = k_{f,2} \cdot filopodia(1 - \frac{VEGFR2aa^{k_{f,3}}}{VEGFR2aa^{k_{f,3}} + k_{f,4}^{k_{f,3}}}) \tag{S36}$$

$$v^{filopodia}_{basalRetraction} = k_{f,5} \cdot filopodia \tag{S37}$$

$$v^{dll4\_mRNA}_{transcription} = k_{+dll4\_mRNA}(\frac{1}{k_{dll4,a}} + (1 - \frac{1}{k_{dll4,a}})\frac{VEGFR2aa^h}{VEGFR2aa^h + k_{dll4,M0}}) \tag{S38}$$

$$v^{dll4\_mRNA}_{degradation} = dll4\_mRNA \cdot k_{-mRNA} \tag{S39}$$

$$v^{notch\_mRNA}_{transcription} = k_{+notch\_mRNA}(\frac{1}{k_{notch,a}} + (1 - \frac{1}{k_{notch,a}})\frac{NICD^h}{NICD^h + k_{notch,M0}}) \tag{S40}$$

$$v^{notch\_mRNA}_{degradation} = notch\_mRNA \cdot k_{-mRNA} \tag{S41}$$

$$v^{VEGFR1\_mRNA}_{transcription} = k_{+VEGFR1\_mRNA}(\frac{1}{k_{VEGFR1,a}} + (1 - \frac{1}{k_{VEGFR1,a}})\frac{NICD^h}{NICD^h + k_{VEGFR1,M0}}) \tag{S42}$$

$$v^{VEGFR1\_mRNA}_{degradation} = VEGFR1\_mRNA \cdot k_{-mRNA} \tag{S43}$$

$$v^{VEGFR2\_mRNA}_{transcription} = k_{+VEGFR2\_mRNA}(1 - (1 - \frac{1}{k_{VEGFR2,a}})\frac{NICD^h}{NICD^h + k_{VEGFR2,M0}}) \tag{S44}$$

$$v^{VEGFR2\_mRNA}_{degradation} = VEGFR2\_mRNA \cdot k_{-mRNA} \tag{S45}$$

3.3 Algebraic Equations

$$s\_range = filopodia/filofactor + 1 \quad \text{scales } filopodia \text{ to distance} \tag{S46}$$

$$VEGF_{here} = \text{VEGF-A in range } s\_range \tag{S47}$$

$$VEGF_{nb} = \text{VEGF-A in range } 1 \tag{S48}$$

$$Dll4_{nb} = \text{dll4 of neighboring cells} \tag{S49}$$

$$VEGFR2aa = VEGFR2a + VEGFR2b \tag{S50}$$

$$\begin{aligned} tip = &(VEGFR2\_mRNA > VEGFR1\_mRNA) \\ &\wedge (filopodia > t_{filo}) \\ &\wedge (dll4\_mRNA > t_{dll4}) \quad \text{tip cell criteria} \end{aligned} \tag{S51}$$

3.4 Equations of Interactions With External Variables

During the update of each agent, VEGF-A is removed from all patches (x, y) in $s - range$ according to

$$min(VEGFA(x, y, t), v^{VEGFR2}_{vegf\_binding} - v^{VEGFR2b}_{dissoc}) \quad \text{interaction with VEGFR2} \tag{S52}$$

$$min(VEGFA(x, y, t), v^{mVEGFR1}_{vegf\_binding} - v^{mVEGFR1b}_{dissoc}) \quad \text{interaction with mVEGFR1} \tag{S53}$$

during the update of each agent.

Similarly, sVEGFR1 is secreted to the neighboring fields according to the value of

$$v^{sVEGFR1}_{secrection} \tag{S54}$$

during the update of each agent.

4 EQUATIONS OF EXTERNAL VARIABLES

Parameter variables are given in Supplemental Tables S1 and S3. Extracellular variables (called local variables in AngioABM) VEGF-A, sVEGFR1, and sVEGFR1b (sVEGFR1 bound to VEGF-A) diffuse, degrade, associate, and dissociate per lattice site (given by (x, y)) according to the rate equations given below:

$$
\begin{aligned}
\frac{\mathrm{d}VEGFA(x,y,t)}{\mathrm{d}t} =& - k_{-VEGFA,ext} \cdot VEGFA(x,y,t) && \text{(S55)} \\
& - k_{sR1:V} \cdot VEGFA(x,y,t) \cdot sVEGFR1(x,y,t) \\
& + k_{sR--V} \cdot sVEGFR1b(x,y,t) \\
& + k_{influx} \cdot (VEGF_{ext,0} - VEGFA(x,y,t)) \\
& + D_{VEGFA} \nabla^2 VEGFA(x,y,t) \\
\frac{\mathrm{d}sVEGFR1(x,y,t)}{\mathrm{d}t} =& - k_{-sVEGFR1,ext} \cdot sVEGFR1(x,y,t) && \text{(S56)} \\
& - k_{sR1:V} \cdot VEGFA(x,y,t) \cdot sVEGFR1(x,y,t) \\
& + k_{sR--V} \cdot sVEGFR1b(x,y,t) \\
& + D_{sVEGFR1} \nabla^2 VEGFR1(x,y,t) \\
\frac{\mathrm{d}sVEGFR1b(x,y,t)}{\mathrm{d}t} =& - k_{-sVEGFR1b,ext} \cdot sVEGFR1b(x,y,t) && \text{(S57)} \\
& + k_{sR1:V} \cdot VEGFA(x,y,t) \cdot sVEGFR1(x,y,t) \\
& - k_{sR--V} \cdot sVEGFR1b(x,y,t) \\
& + D_{sVEGFR1b} \nabla^2 sVEGFR1b(x,y,t) \\
& && \text{(S58)}
\end{aligned}
$$

Notice that the secretion of sVEGFR1 occurs during updates of agents.

5 TABLES OF PARAMETERS

The tables listed here are intended to give an overview of the parameters used. A full table with additional information is available as Supplemental Table S3 in Microsoft Excel Open XML Format.

parameter	function	value	unit	literature
$filopodia$	initial value	54	$a.u.$	
$k_{f,1}$	rate of filopodia extension	0.6	$1/h$	
$k_{f,2}$	max. rate of act. retraction	0.8	$1/h$	
$k_{f,3}$	Hill exponent	2		
$k_{f,4}$	Km	300	$molecules$	
$k_{f,5}$	rate of basal retraction	0.4	$1/h$	
$filofactor$	scaling factor	400		
t_{filo}	tip cell marker	250	$a.u.$	
t_{dll4}	tip cell marker	13	$molecules$	
$VEGFA_{ext,0}$	initial value	5200	$molecules$	Wang et al. (2002)<br>Ubezio et al. (2016)<br>Bentley et al. (2008)<br>Mac Gabhann and Popel (2006)<br>Shamloo et al. (2012)
$sVEGFR1_{free,0}$	initial value	0	$molecules$	
$sVEGFR1_{bound,0}$	initial value	0	$molecules$	
D_{VEGFA}	diffusion coefficient	2200	$\mu m^2/h$	Köhn-Luque et al. (2013)<br>Chen et al. (2007)<br>Gabhann et al. (2006)<br>Vempati et al. (2010)
$D_{sVEGFR1}$	diffusion coefficient	1800	$\mu m^2/h$	Hashambhoy et al. (2011)
$D_{sVEGFR1-VEGFA}$	diffusion coefficient	990	$\mu m^2/h$	
$k_{-VEGFA,ext}$	degradation rate	$2.5 \cdot 10^{-4}$	$1/h$	
$k_{-sVEGFR1_{free},ext}$	degradation rate	0.28	$1/h$	
$k_{-sVEGFR1_{bound},ext}$	degradation rate	0.04	$1/h$	
$k_{sR1:V}$	binding constant	0.65237	$\frac{1}{h \cdot molecules}$	
k_{sR--V}	dissociation constant	0.4	$1/h$	
k_{influx}	VEGF influx parameter	0.25	$\frac{1}{h \cdot latticesite}$	

Table S1. Parameters of the agent based model. Parameter names as used in the List of Equations. A.u. indicates arbitrary units.

Table S2: Parameters used in intracellular signaling. For some parameters, values are sampled from log-normal distributions for each cell. Column 'sd' indicates the standard deviation of these distributions. If no standard deviation is indicated, fixed values are used. For the protein and mRNA degradation rates, the maximal transcription rates and the translation rates, we have referred to Schwanhäusser et al. (2011).

name	function	mean	sd	unit	literature
$dll4_0$	initial value	87	4.35	particles	Bentley et al. (2008)
					Carlier et al. (2012)
$dll4mRNA_0$	initial value	6	0.3	particles	Schwanhausser (2011)
$mVEGFR1_0$	initial value	1075	53.75	particles	Imoukhuede et al. (2013)
					Gabhann and Popel (2006)
					Carlier et al. (2012)
$mVEGFR1b_0$	initial value	6718	335.9	particles	
$nicd_0$	initial value	494	24.7	particles	
$notch_0$	initial value	258	12.9	particles	Bentley et al. (2008)
					Boareto et al. (2015)
$notchmRNA_0$	initial value	21	1.05	particles	
$sVEGFR1_0$	initial value	731	36.55	particles	Imoukhuede et al. (2013)
					Gabhann and Popel (2006)
					Carlier et al. (2012)
$VEGFR1mRNA_0$	initial value	29	1.45	particles	
$VEGFR2_0$	initial value	940	47	particles	Imoukhuede et al. (2013)
					Bentley et al. (2008)
					Bentley et al. (2009)
					Gabhann et al. (2006)
$VEGFR2b_0$	initial value	47	2.35	particles	
$VEGFR2mRNA_0$	initial value	6	0.3	particles	
$VEGFR2a_0$	initial value	48	2.4	particles	
h	Hill coefficient	2			Venkatraman et al. (2016)
					Boareto et al. (2015)
$k_{dll4,a}$	max. fold increase	16			Ubezio et al. (2016)
					Bentley et al. (2008)
$k_{dll4,M0}$	K_M of Hill-fct	450		particles	
k_{-mRNA}	degradation rate	0.1		1/h	Schwanhäusser et al. (2011)
					Yang et al. (2003)
k_{-Dll4}	protein deg.	0.73		1/h	Schwanhäusser et al. (2011)
$k_{-mVEGFR1}$	protein deg.	0.48		1/h	
k_{-Notch}	protein deg.	0.52		1/h	
$k_{-VEGFR2}$	protein deg.	0.24		1/h	
$k_{-VEGFR2a}$	protein deg.	0.1		1/h	
$k_{-sVEGFR1}$	protein deg.	0.65		1/h	
k_{-NICD}	protein deg.	0.48	0.024	1/h	
$k_{sVEGFR1,export}$	secretion rate	0.64		1/h	Walpole et al. (2015)
					Wu et al. (2010)
$k_{mR1:V}$	association rate	$5 \cdot 10^{-7}$		$\frac{1}{h \cdot particles}$	Mac Gabhann and Popel (2006)

Table S2: Parameters used in intracellular signaling. For some parameters, values are sampled from log-normal distributions for each cell. Column 'sd' indicates the standard deviation of these distributions. If no standard deviation is indicated, fixed values are used. For the protein and mRNA degradation rates, the maximal transcription rates and the translation rates, we have referred to Schwanhäusser et al. (2011).

name	function	mean	sd	unit	literature
$k_{mR2:V}$	association rate	$3.76 \cdot 10^{-7}$		$\frac{1}{h \cdot particles}$	Cunningham et al. (2000)
k_{R--V}	dissociation rate	0.4		1/h	Cunningham et al. (2000)
$k_{\sim VEGFR2a}$	inactivation rate	0.2		1/h	
k_c	cis inhibition rate	0.27		$\frac{1}{h \cdot particles}$	Sprinzak et al. (2011)
k_t	trans activation rate	0.14		$\frac{1}{h \cdot particles}$	
$k_{notch,a}$	max fold increase	5			
$k_{notch,M0}$	K_M of Hill-fct	350		particles	
$k_{VEGFR1,a}$	max fold increase	19.93			Harrington et al. (2008)
$k_{VEGFR1,M0}$	K_M of Hill-fct	250		particles	
$k_{VEGFR2,a}$	max fold increase	37.57			Bentley et al. (2008) Harrington et al. (2008)
$k_{VEGFR2,M0}$	K_M of Hill-fct	250		particles	
k_{+dll4_mRNA}	max transcription rate	6	0.3	1/h	Schwanhäusser et al. (2011)
k_{+notch_mRNA}	max transcription rate	2.5	0.13	1/h	
$k_{+VEGFR1\_mRNA}$	max transcription rate	23	2.3	1/h	
$k_{+VEGFR2\_mRNA}$	max transcription rate	2.83	0.14	1/h	
k_{+Dll4}	translation rate	80	4	1/h	
$k_{+mVEGFR1}$	translation rate	24.3	1.22	1/h	
k_{+Notch}	translation rate	26	1.3	1/h	
$k_{+sVEGFR1}$	translation rate	36.45	1.8225	1/h	
$k_{+VEGFR2}$	translation rate	38.791	1.93955	1/h	

6 PARAMETER SENSITIVITY - RELATIVE VESSEL AREA

For each parameter of the agent based model, we computed 20 simulations for 27 different parameter values between 0.75x and 1.25x the original parameter value. The relative vessel area, A_{EC}^{rel} was recorded as model output. Plots indicate mean and standard deviation for each parameter value. Parameter names are the same as used in the equations above and Tables S1, S2. In Table S3 these names occur in column E (name in table of equations).

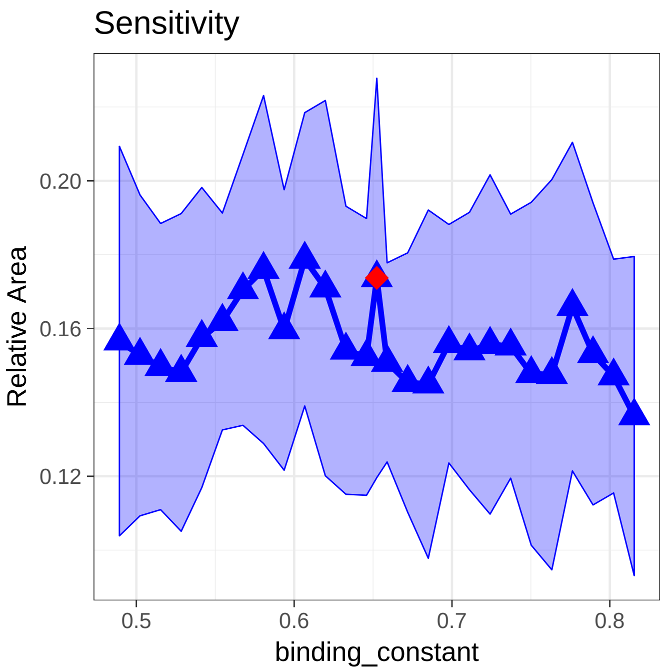

Figure S2: Parameter sensitivity of the binding constant of binding of external sVEGFR to VEGF-A with respect to relative vessel area.

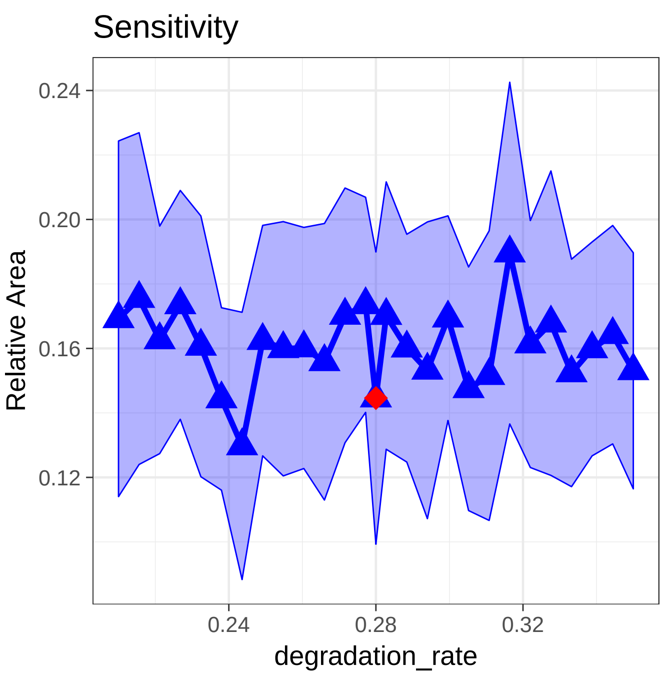

Figure S3: Parameter sensitivity of the extracellular degradation rate of sVEGFR1 with respect to relative vessel area.

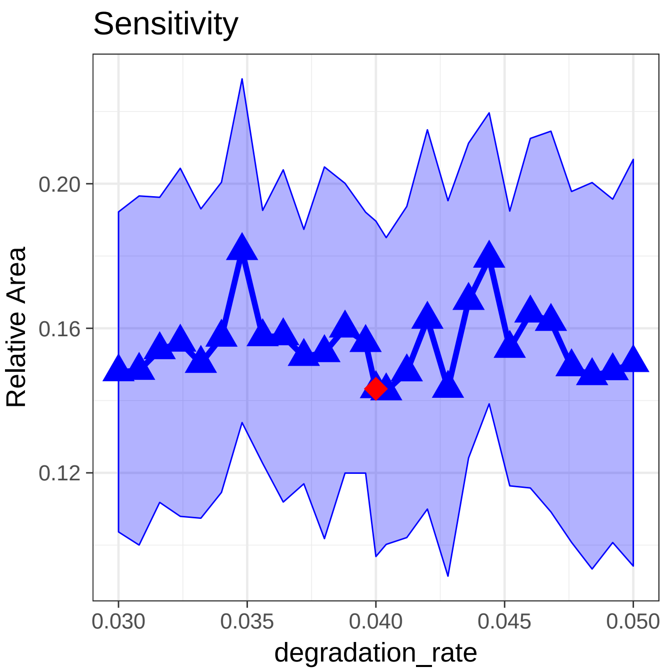

Figure S4: Parameter sensitivity of the extracellular degradation rate of sVEGFR1 bound to VEGF-A with respect to relative vessel area.

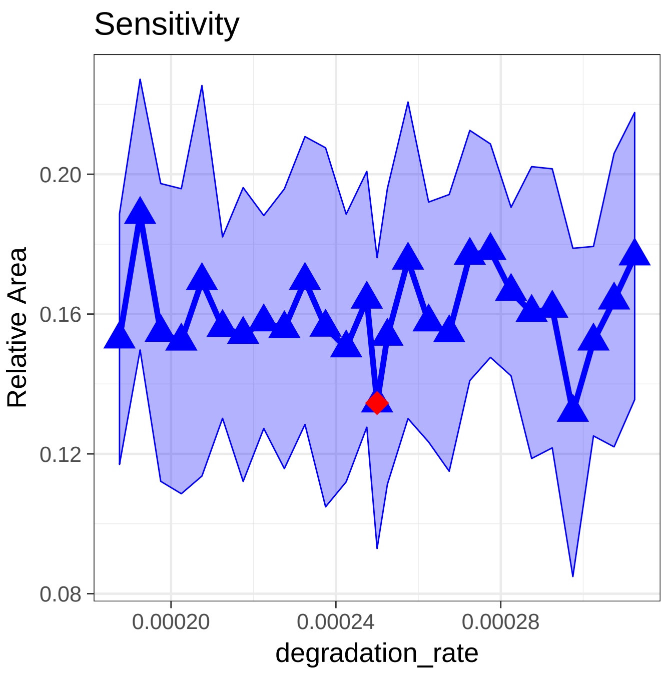

Figure S5: Parameter sensitivity of the extracellular degradation rate of VEGF-A with respect to relative vessel area.

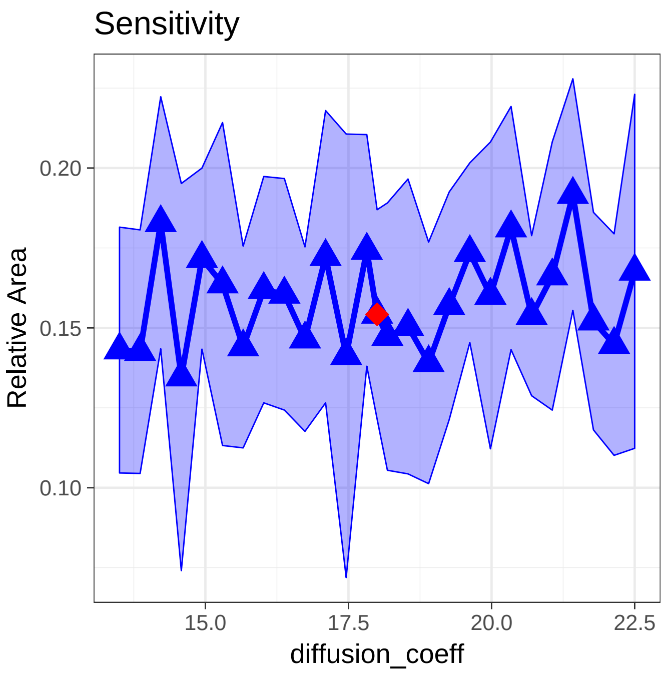

Figure S6: Parameter sensitivity of the diffusion coefficient of sVEGFR1 with respect to relative vessel area.

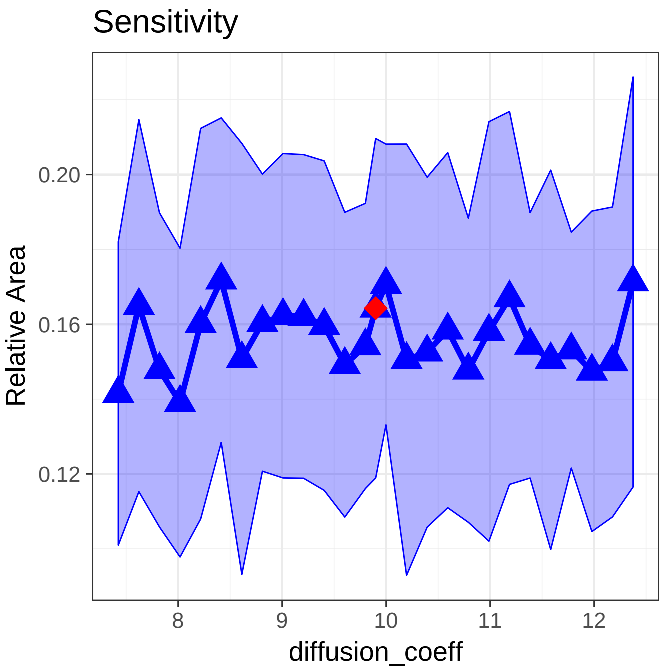

Figure S7: Parameter sensitivity of the diffusion coefficient of sVEGFR1 bound to VEGF-A with respect to relative vessel area.

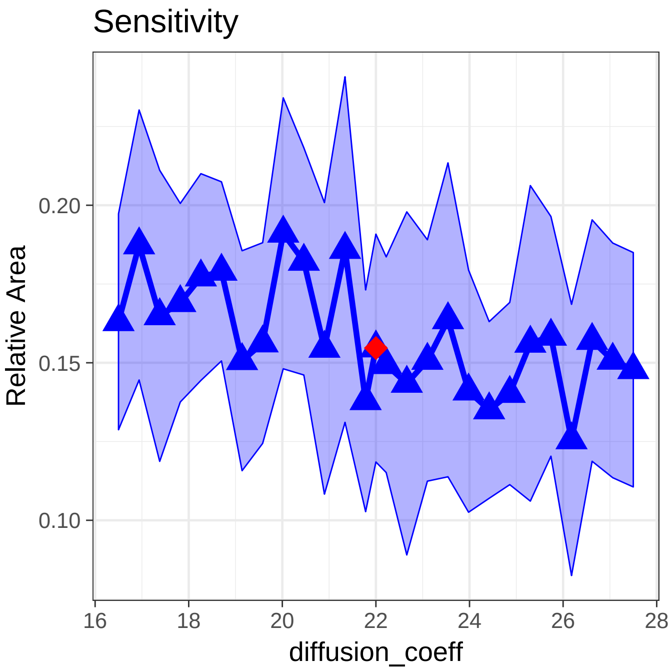

Figure S8: Parameter sensitivity of the diffusion coefficient of VEGF-A with respect to relative vessel area.

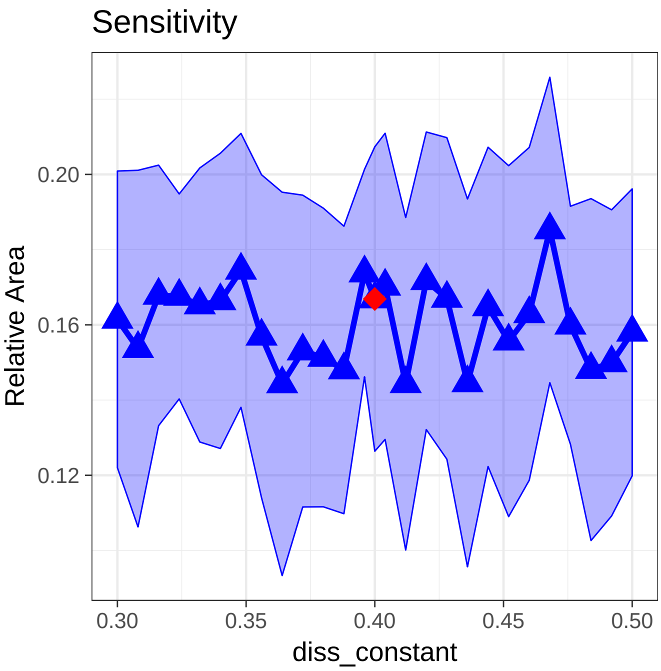

Figure S9: Parameter sensitivity of the dissociation rate of sVEGFR1 bound to VEGF-A with respect to relative vessel area.

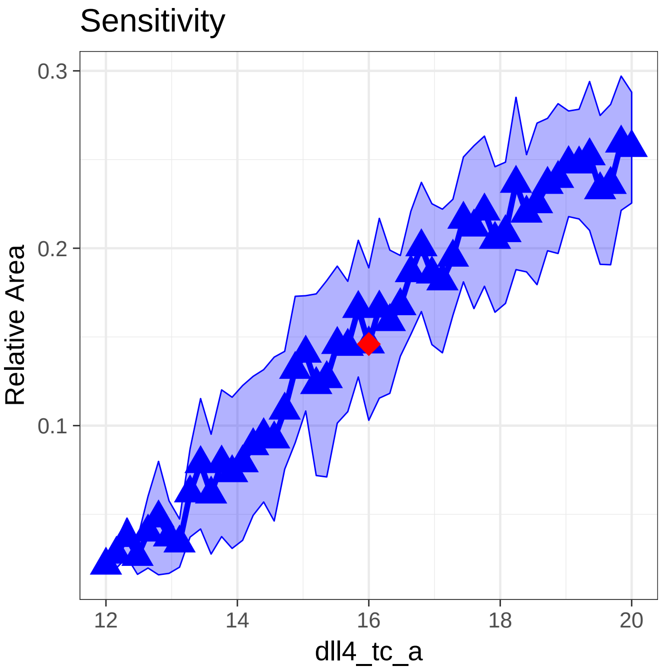

Figure S10: Parameter sensitivity of $k_{dll4,a}$ with respect to relative vessel area.

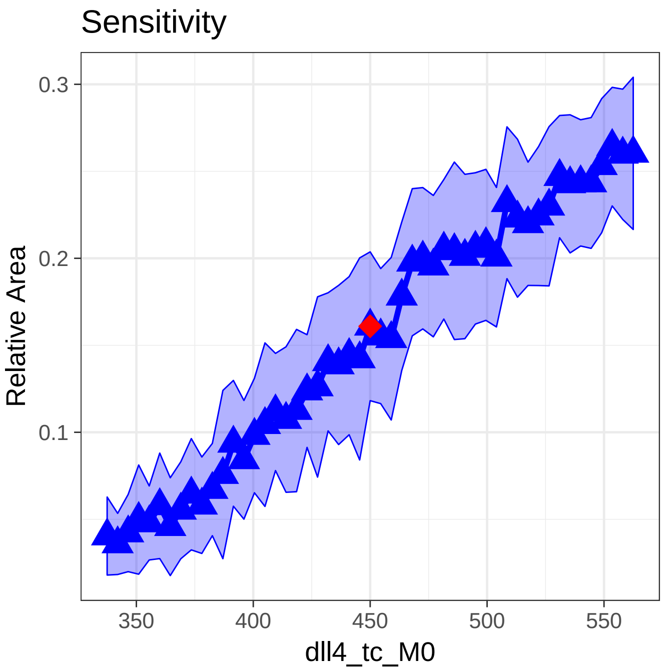

Figure S11: Parameter sensitivity of $k_{dll4,M0}$ with respect to relative vessel area.

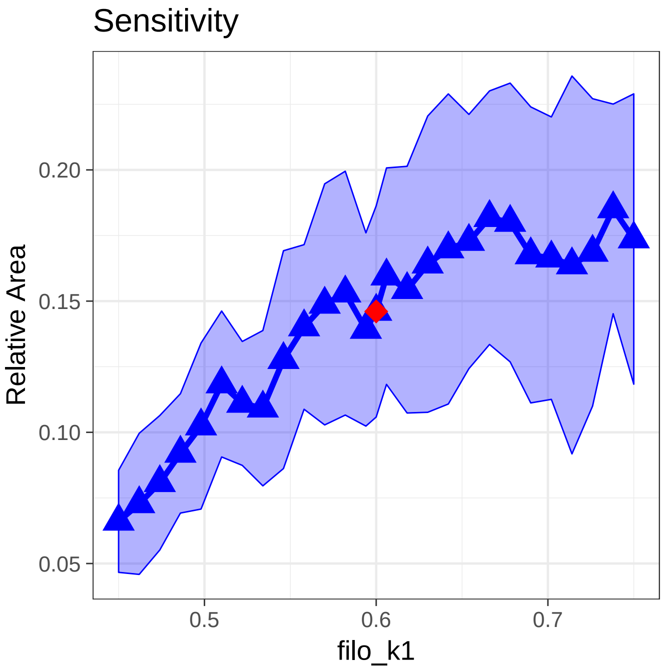

Figure S12: Parameter sensitivity of $k_{f,1}$ with respect to relative vessel area.

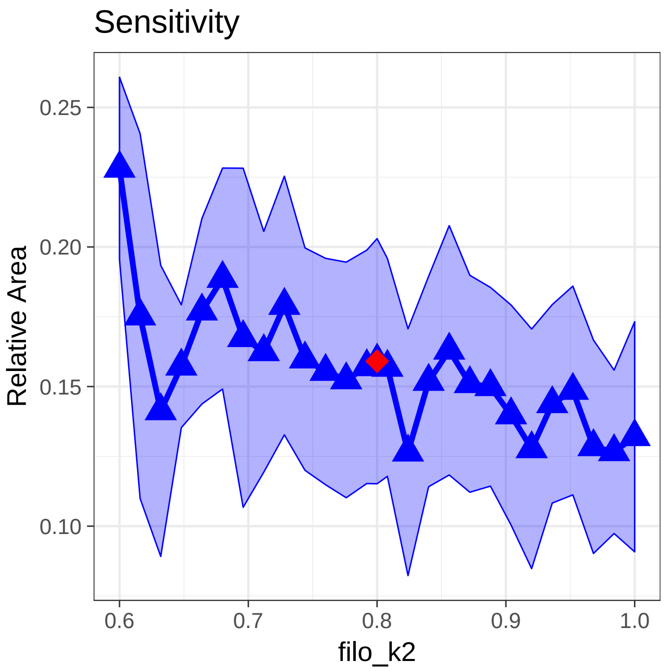

Figure S13: Parameter sensitivity of $k_{f,2}$ with respect to relative vessel area.

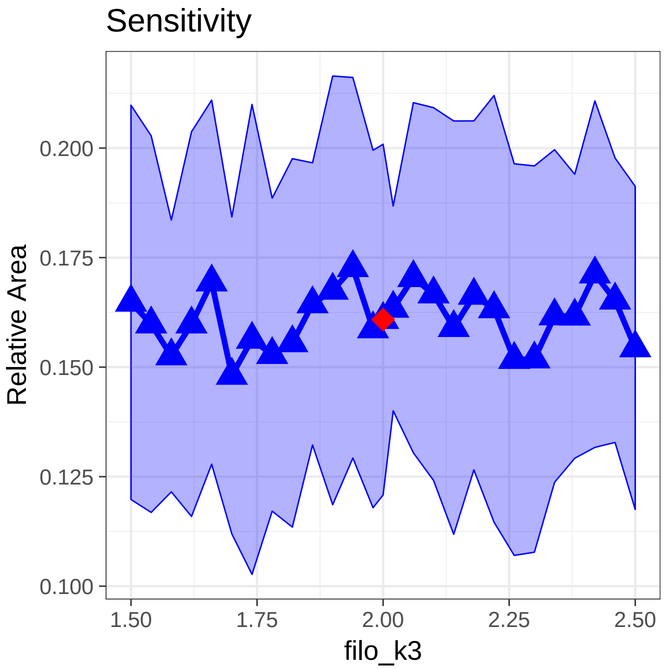

Figure S14: Parameter sensitivity of $k_{f,3}$ with respect to relative vessel area.

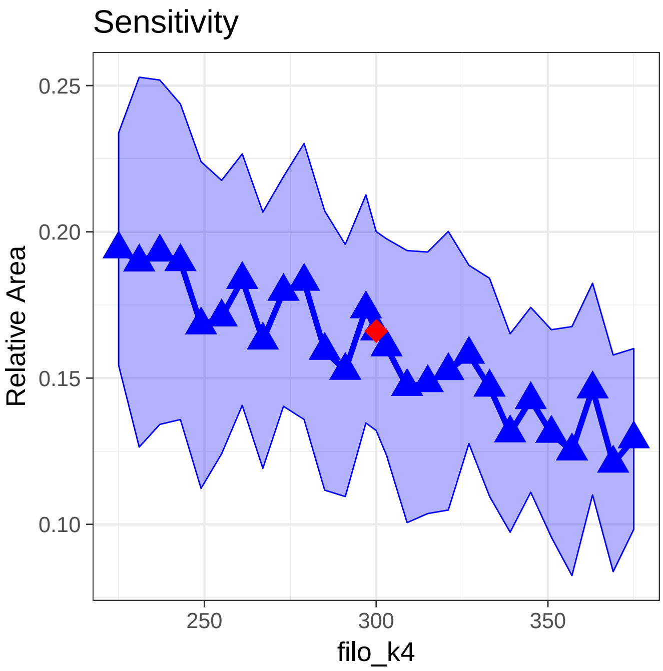

Figure S15: Parameter sensitivity of $k_{f,4}$ with respect to relative vessel area.

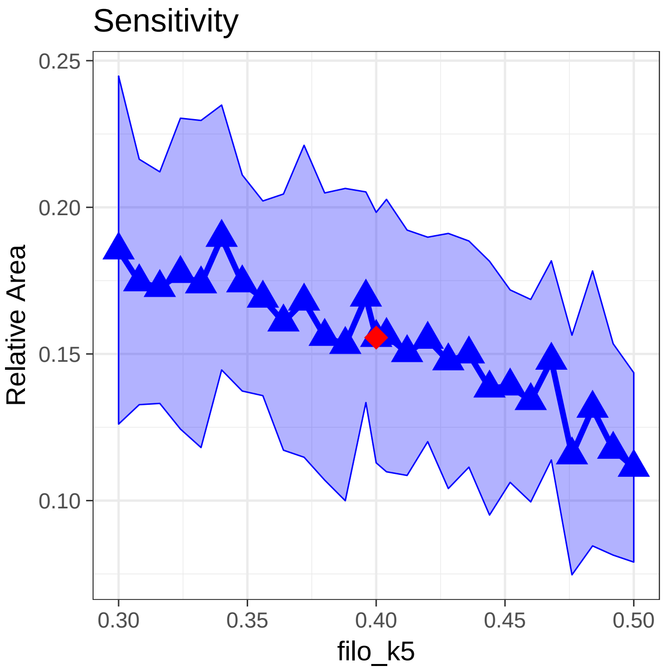

Figure S16: Parameter sensitivity of $k_{f,5}$ with respect to relative vessel area.

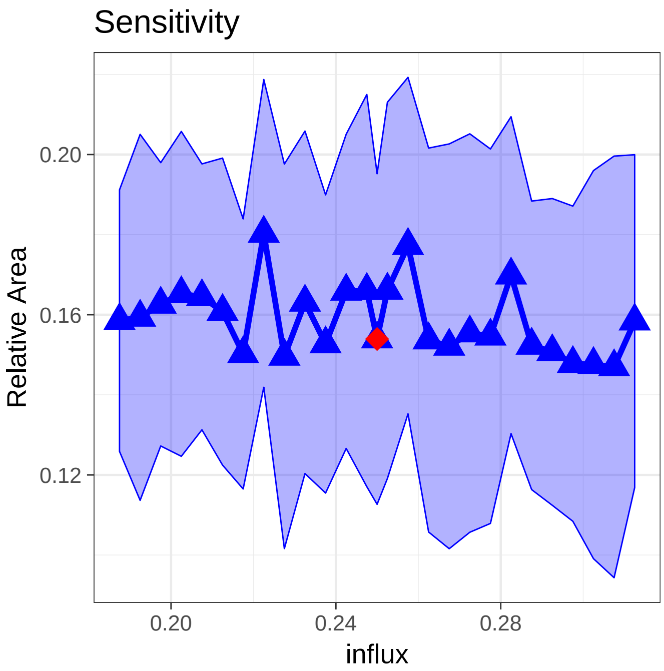

Figure S17: Parameter sensitivity of the influx rate of VEGF-A with respect to relative vessel area.

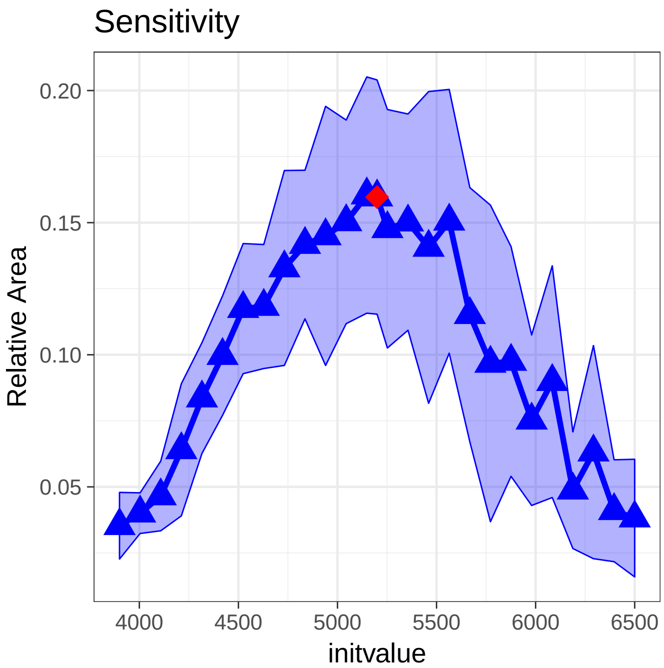

Figure S18: Parameter sensitivity of the reference value of VEGF-A in the medium with respect to relative vessel area.

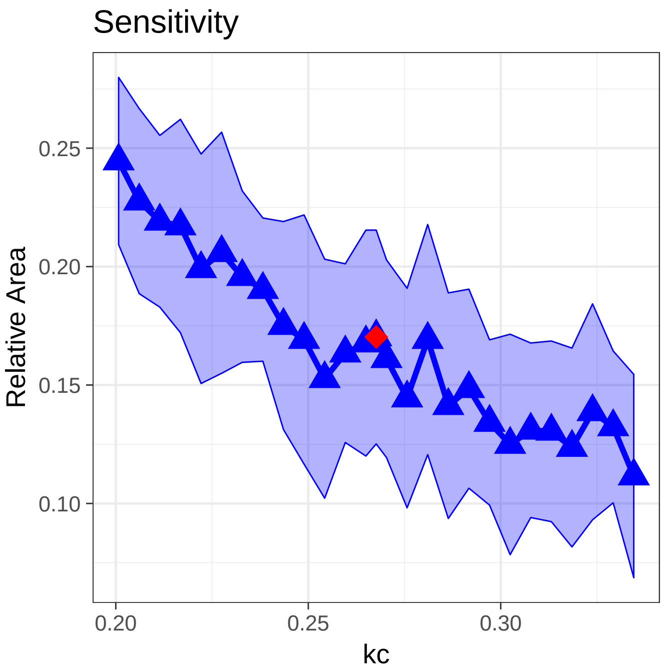

Figure S19: Parameter sensitivity of k_c with respect to relative vessel area.

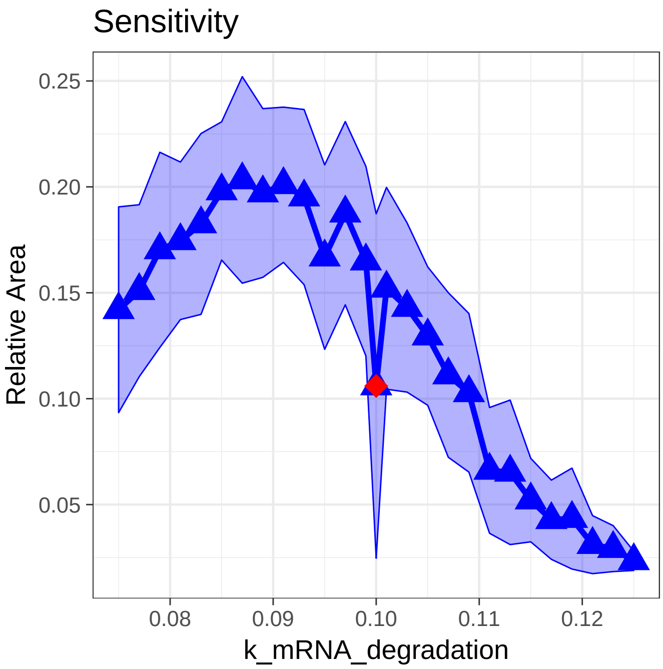

Figure S20: Parameter sensitivity of k_{-mRNA} with respect to relative vessel area.

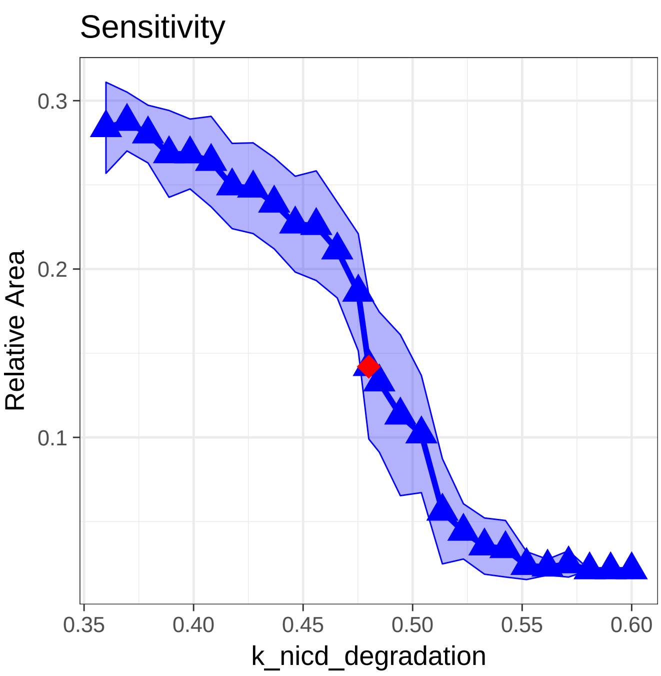

Figure S21: Parameter sensitivity of k_{-NICD} with respect to relative vessel area.

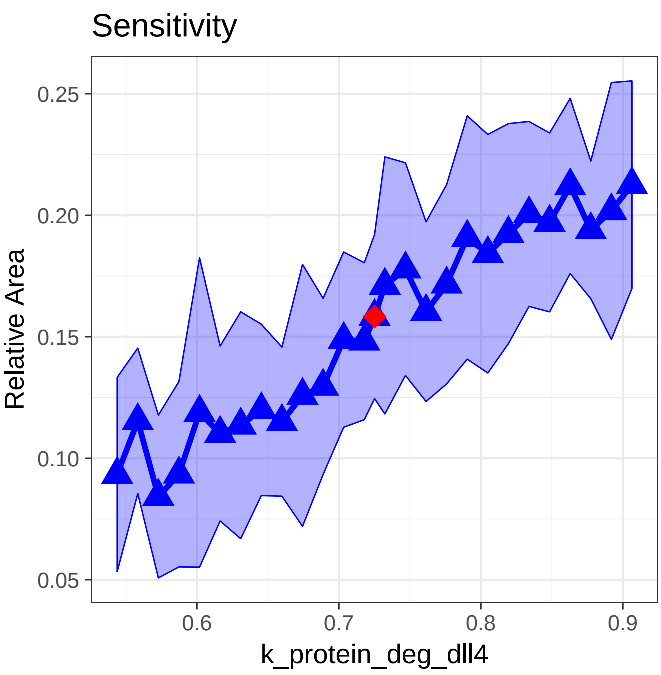

Figure S22: Parameter sensitivity of k_{-Dll4} with respect to relative vessel area.

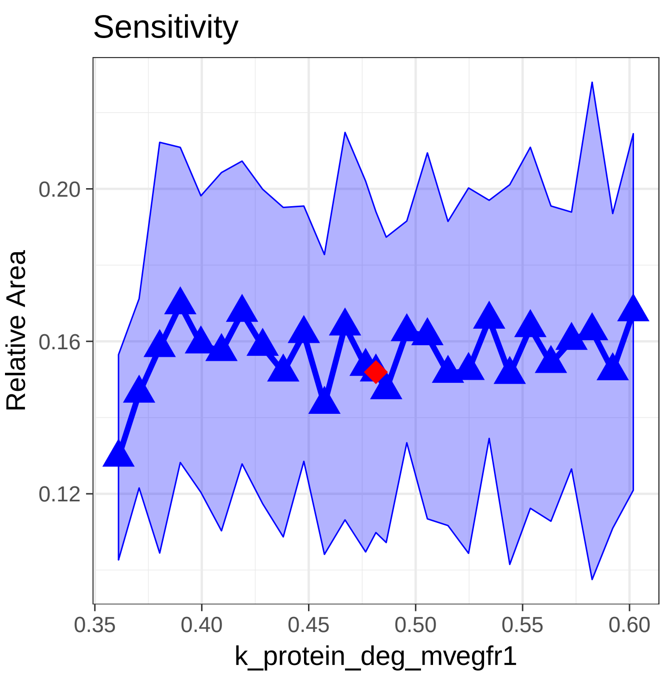

Figure S23: Parameter sensitivity of $k_{-mVEGFR1}$ with respect to relative vessel area.

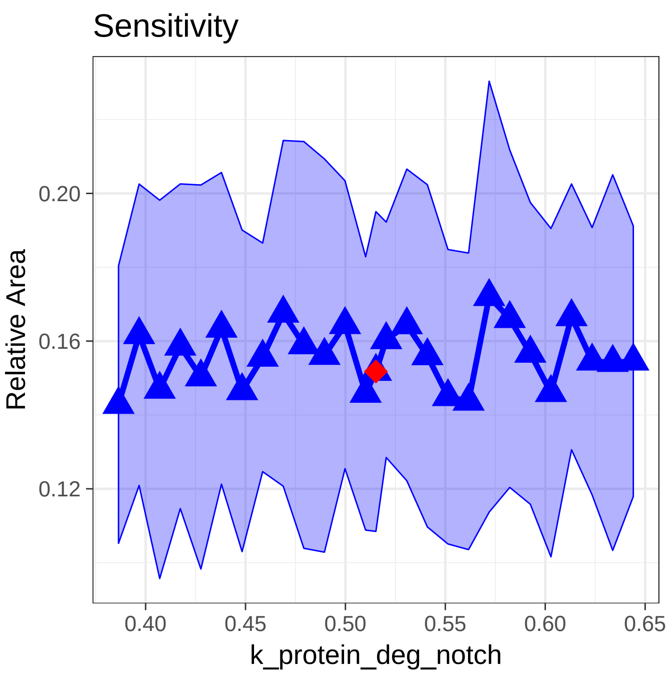

Figure S24: Parameter sensitivity of k_{-Notch} with respect to relative vessel area.

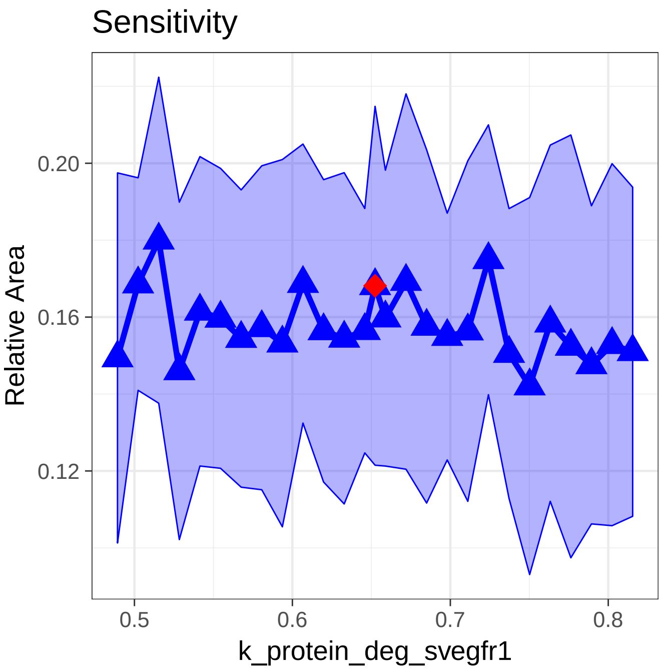

Figure S25: Parameter sensitivity of $k_{-sVEGFR1}$ (the intracellular degradation rate of sVEGFR1) with respect to relative vessel area.

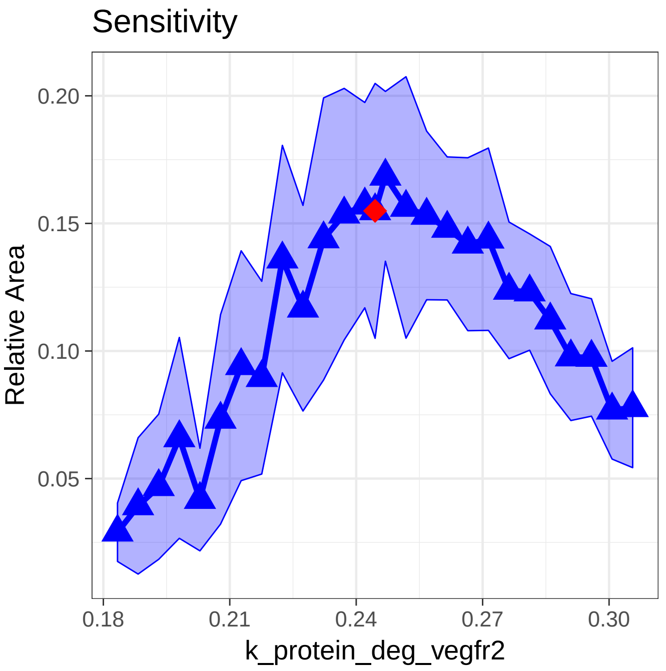

Figure S26: Parameter sensitivity of $k_{-VEGFR2}$ with respect to relative vessel area.

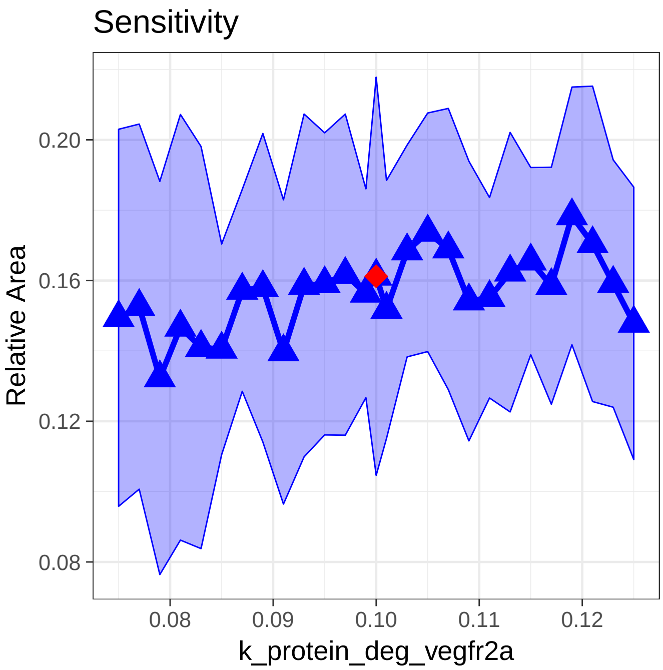

Figure S27: Parameter sensitivity of $k_{-VEGFR2a}$ with respect to relative vessel area.

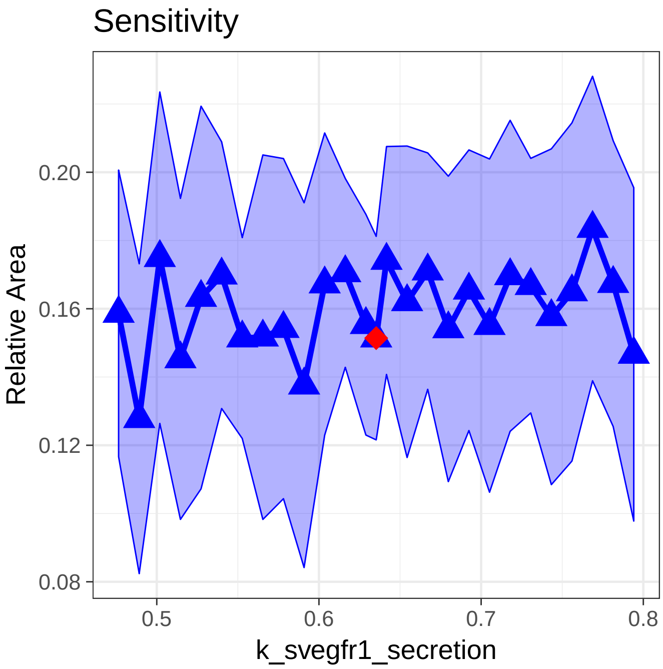

Figure S28: Parameter sensitivity of $k_{sVEGFR1,export}$ with respect to relative vessel area.

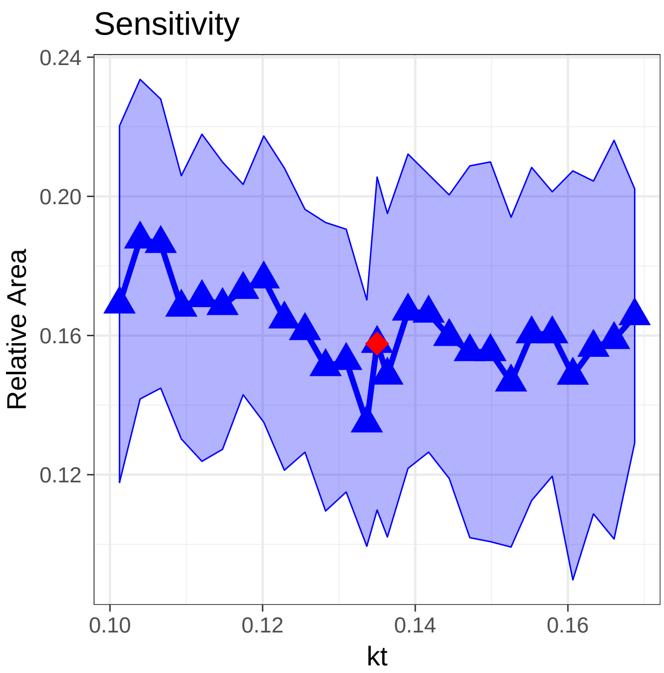

Figure S29: Parameter sensitivity of k_t with respect to relative vessel area.

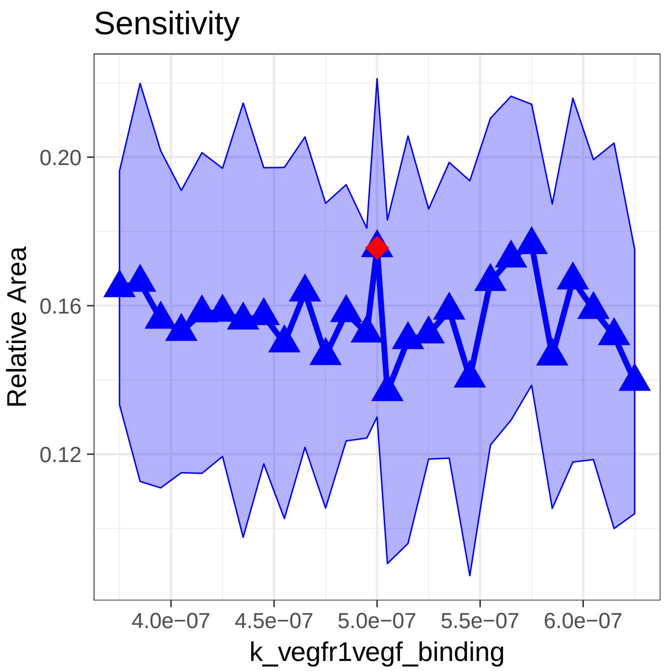

Figure S30: Parameter sensitivity of $k_{mR1:V}$ with respect to relative vessel area.

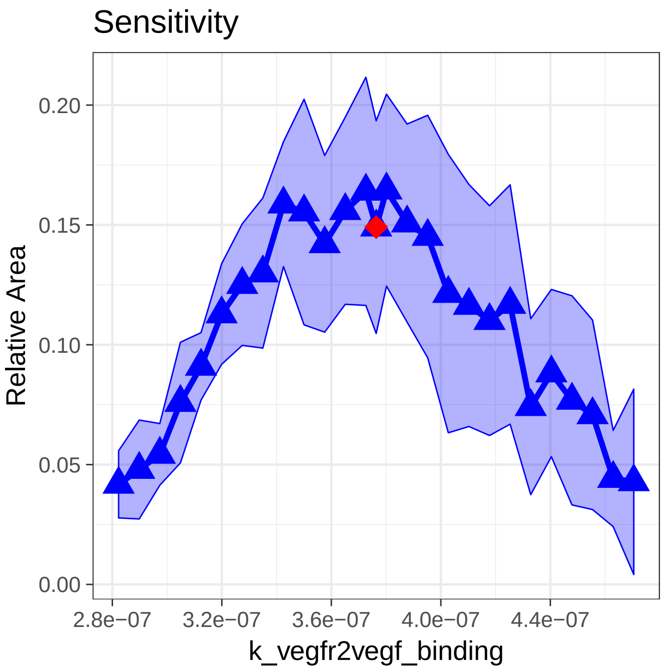

Figure S31: Parameter sensitivity of $k_{mR2:V}$ with respect to relative vessel area.

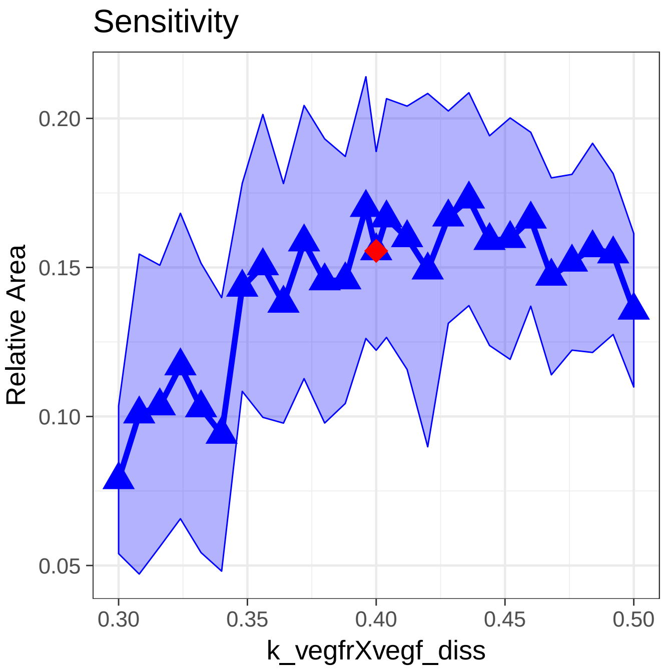

Figure S32: Parameter sensitivity of k_{R--V} with respect to relative vessel area.

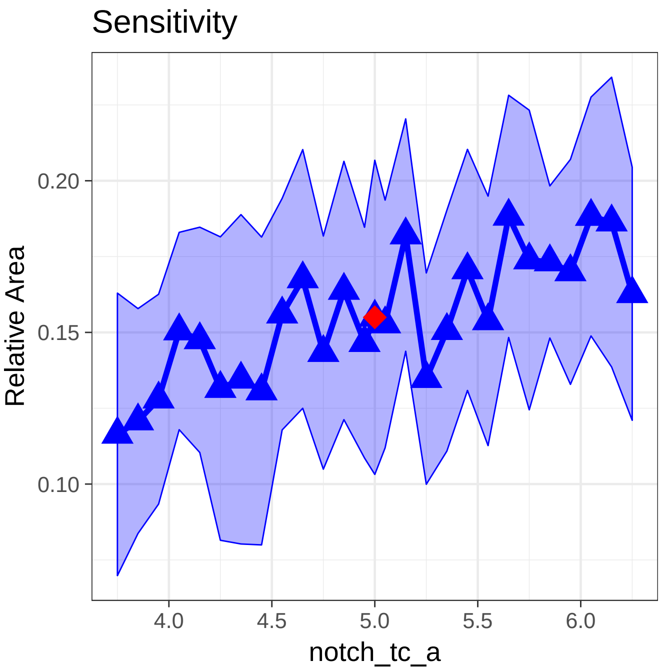

Figure S33: Parameter sensitivity of $k_{notch,a}$ with respect to relative vessel area.

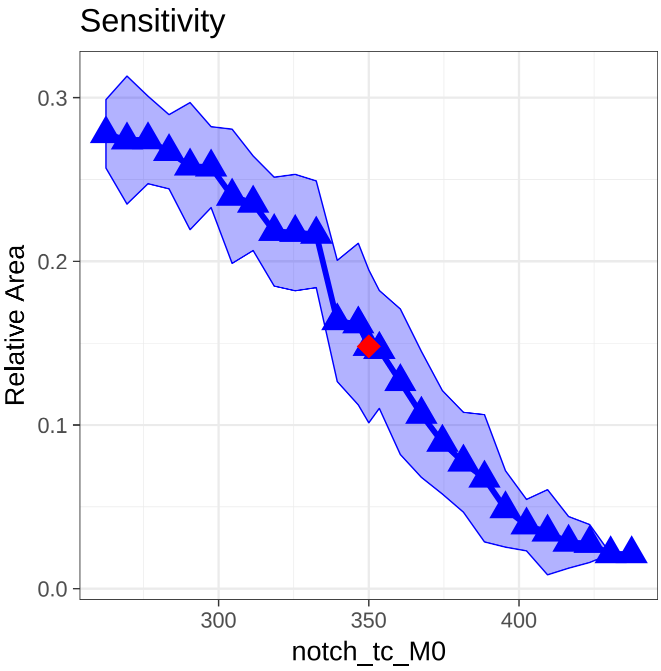

Figure S34: Parameter sensitivity of $k_{notch,M0}$ with respect to relative vessel area.

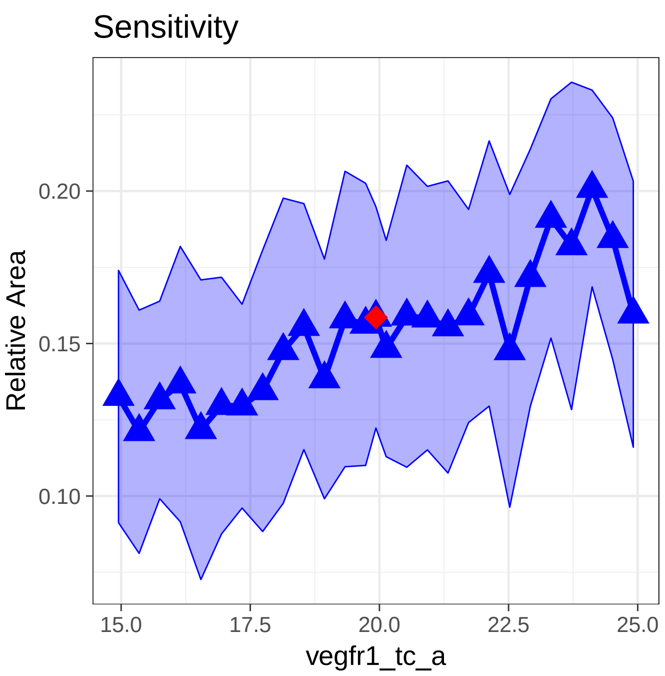

Figure S35: Parameter sensitivity of $k_{VEGFR1,a}$ with respect to relative vessel area.

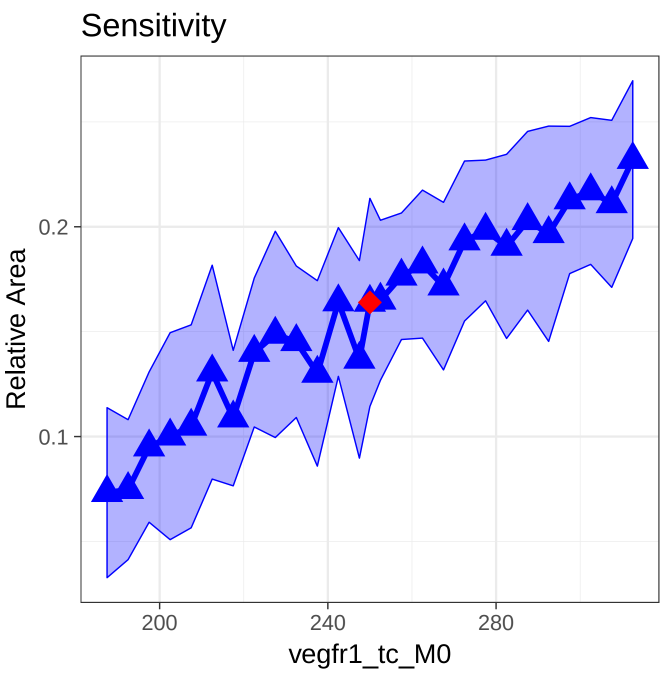

Figure S36: Parameter sensitivity of $k_{VEGFR1,M0}$ with respect to relative vessel area.

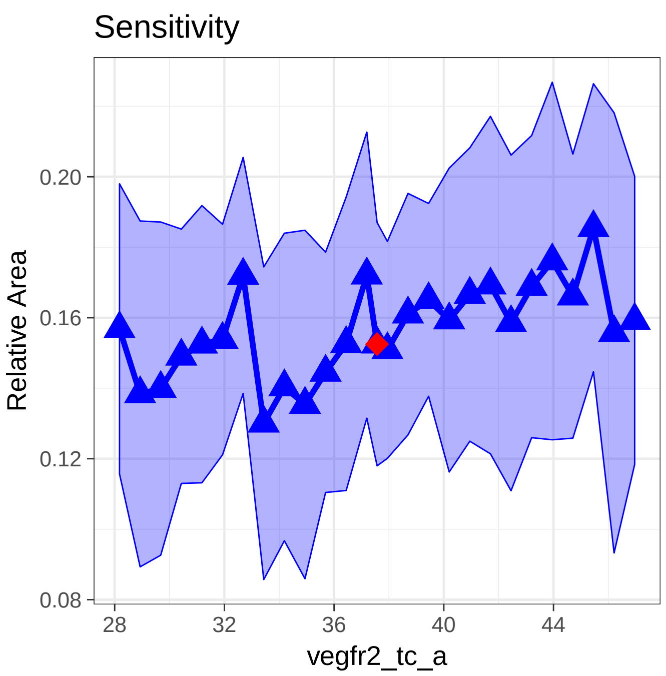

Figure S37: Parameter sensitivity of $k_{VEGFR2,a}$ with respect to relative vessel area.

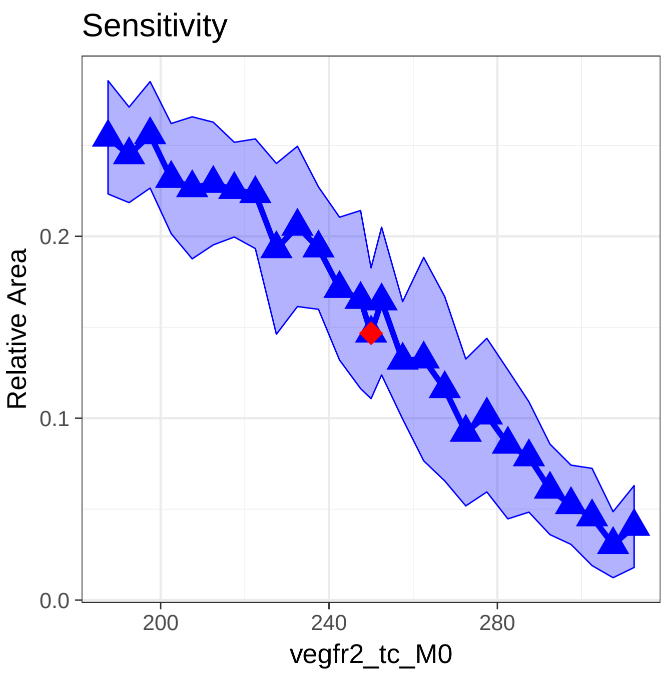

Figure S38: Parameter sensitivity of $k_{VEGFR2,M0}$ with respect to relative vessel area.

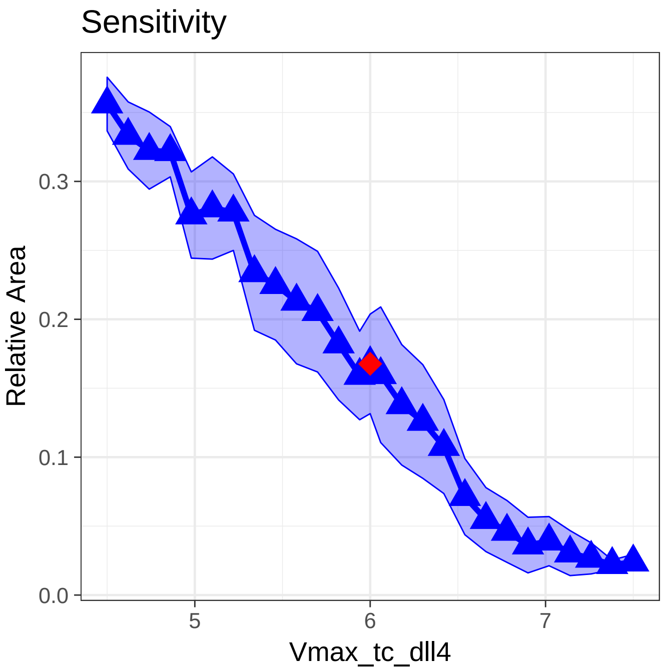

Figure S39: Parameter sensitivity of k_{+dll4_mRNA} with respect to relative vessel area.

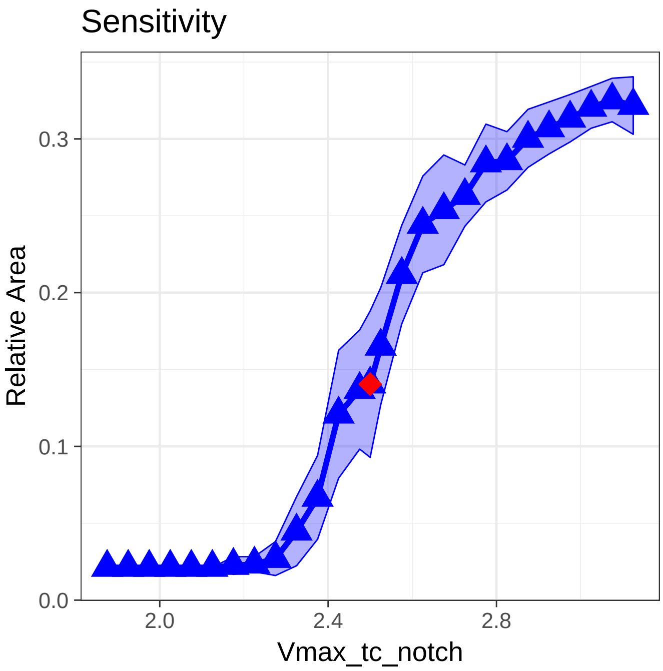

Figure S40: Parameter sensitivity of k_{+notch_mRNA} with respect to relative vessel area.

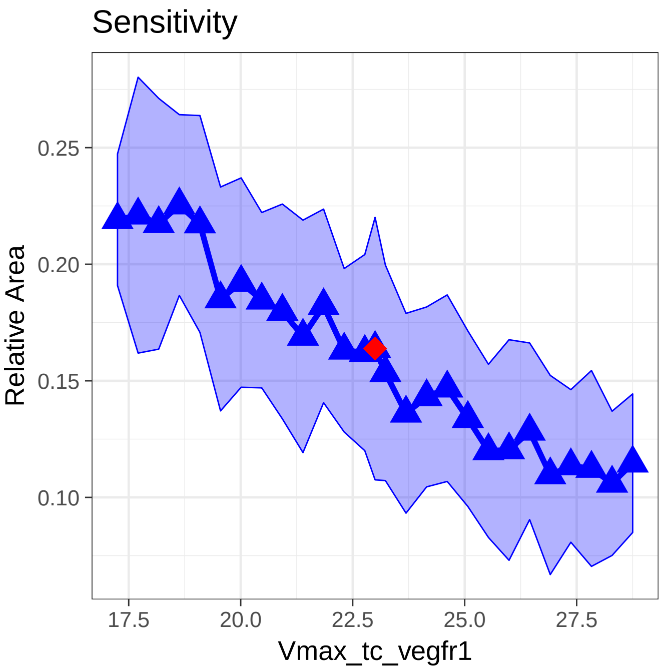

Figure S41: Parameter sensitivity of $k_{+VEGFR1\_mRNA}$ with respect to relative vessel area.

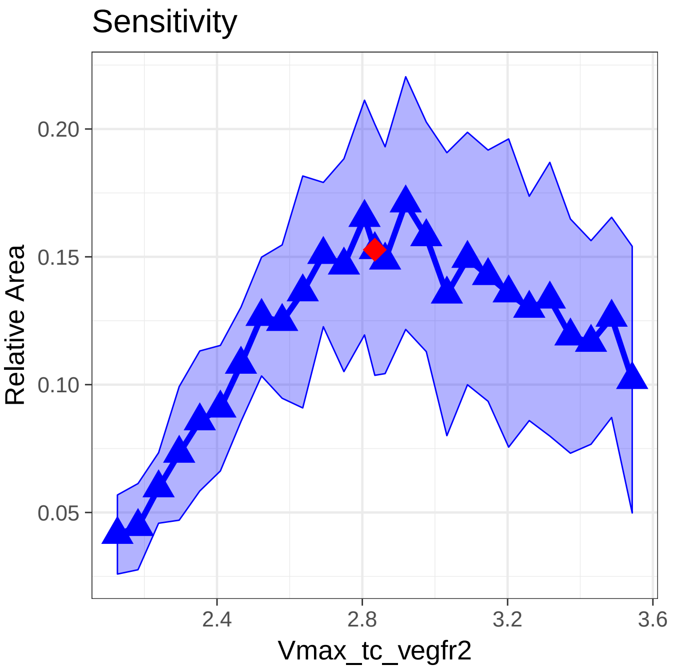

Figure S42: Parameter sensitivity of $k_{+VEGFR2\_mRNA}$ with respect to relative vessel area.

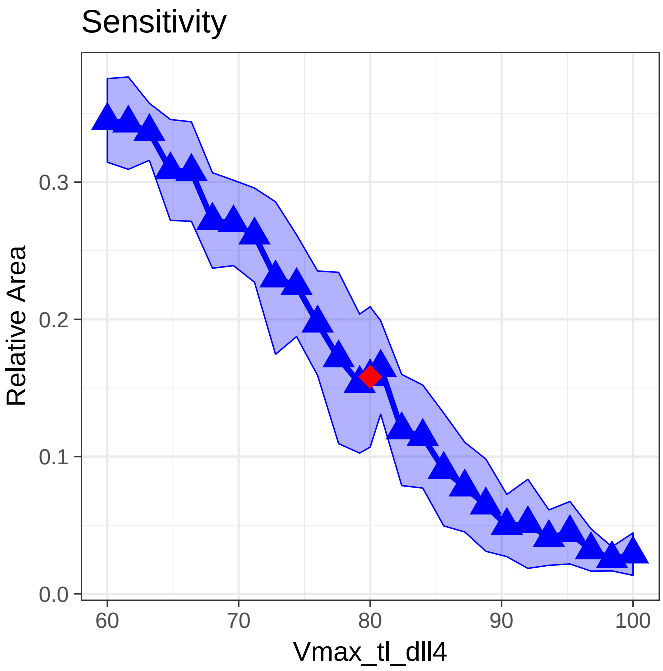

Figure S43: Parameter sensitivity of k_{+Dll4} with respect to relative vessel area.

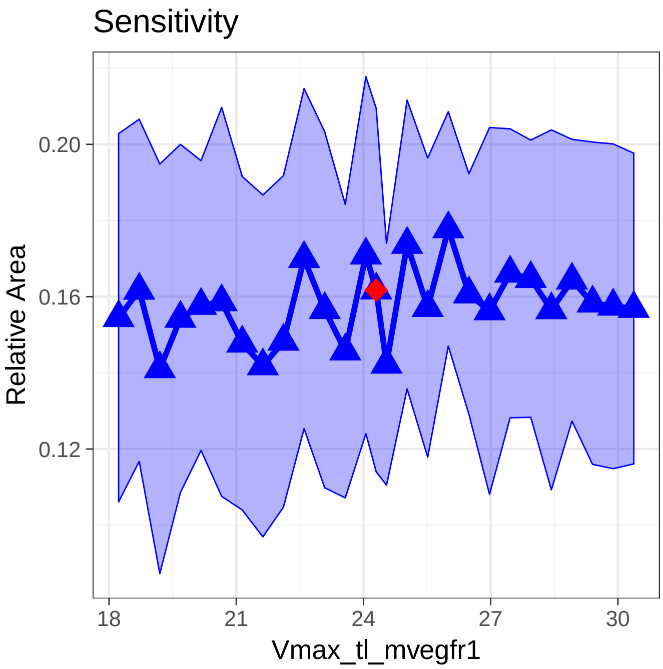

Figure S44: Parameter sensitivity of $k_{+mVEGFR1}$ with respect to relative vessel area.

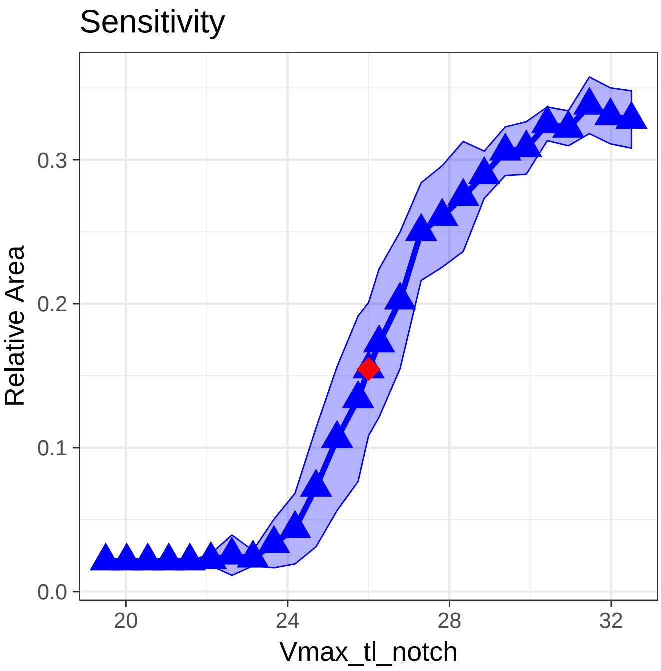

Figure S45: Parameter sensitivity of k_{+Notch} with respect to relative vessel area.

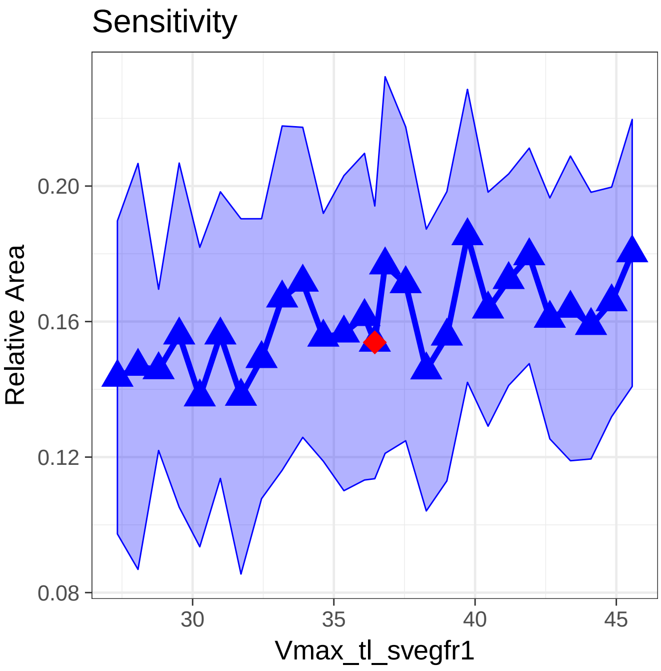

Figure S46: Parameter sensitivity of $k_{+sVEGFR1}$ with respect to relative vessel area.

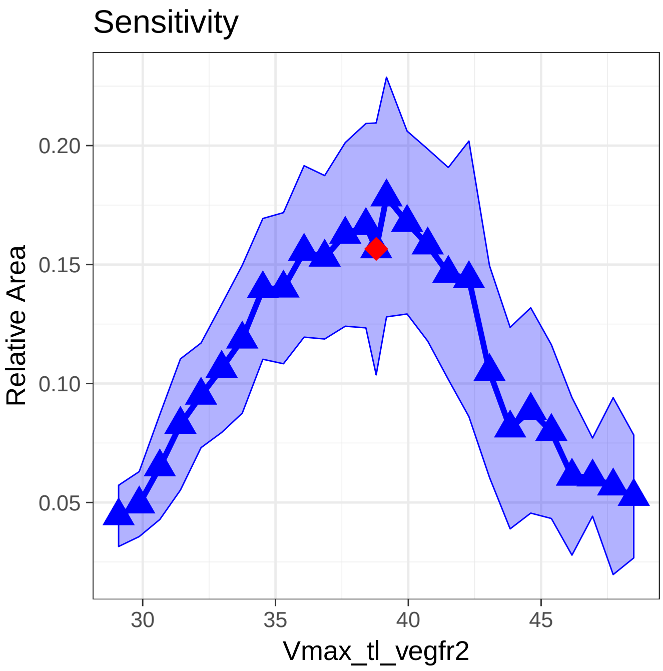

Figure S47: Parameter sensitivity of $k_{+VEGFR2}$ with respect to relative vessel area.

7 PARAMETER SENSITIVITY - BRANCH POINTS

For each parameter analyzed in Figures 4 and 5 of the main text, we computed branch points/mm. As in the Figures 4 and 5, we used 30 simulations for 51 different parameter values between 0x and 2x the original parameter value.

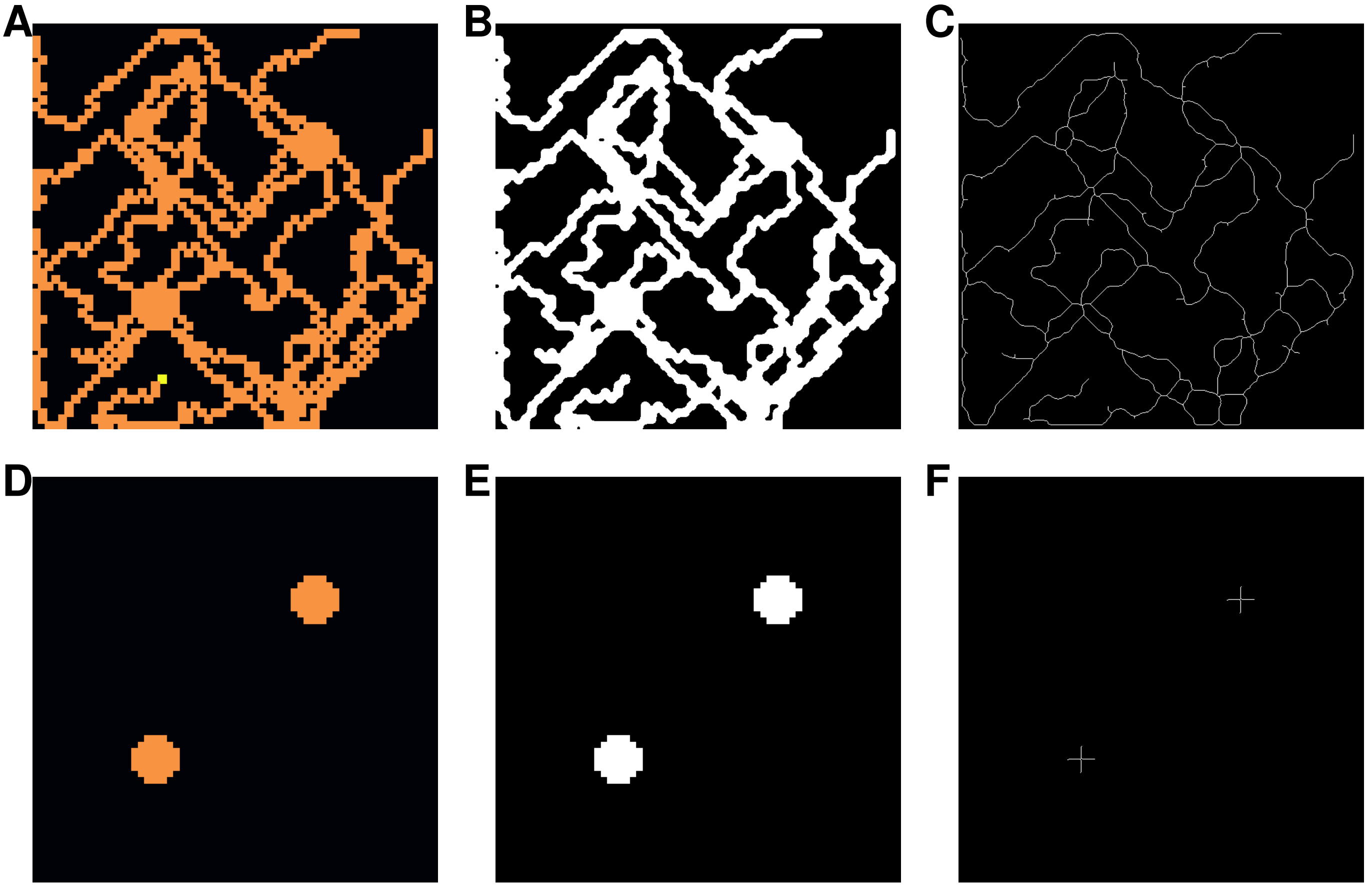

Figure S48: Work flow for measuring branch points from agent based model simulations. A: plot of simulation results with sprouting, B: image from A smoothened and binarized, C: skeletonized version of B. D: plot of simulation results without sprouting, E: image from D smoothened and binarized, F: skeletonized version of E. Note that very short endpoints in the skeleton are excluded from branch point analysis.

Branch points/mm have been computed as described in the main text. To measure branch points, snapshots of the simulation representing $600\mu m$ x $600\mu m$ were saved as tiff image at a resolution of 600 x 600 pixels. These were analyzed with ImageJ-MATLAB Hiner et al. (2017). Images were smoothened to ensure connectivity of agents, then thresholded, binarized and skeletonized. The skeletonized path was analyzed for branch points using AnalzyeSkeleton Arganda-Carreras et al. (2010). Figure S48 A - C displays an overview of this process. Branch points per millimeter vessel length were computed using the total length of all branches from the ImageJ analysis. The minimum of 0 and branch points - 5 was used to correct for artificial branch points that arise from skeletonizing the initial cell populations in the absence of any sprouting or with very low sprouting, as indicated in Figure S48, D - F.

Plots indicate mean and standard deviation for each parameter value. Parameter names are the same as used in the equations above, in the table of parameter values, these names occur in column E (name in table of equations).

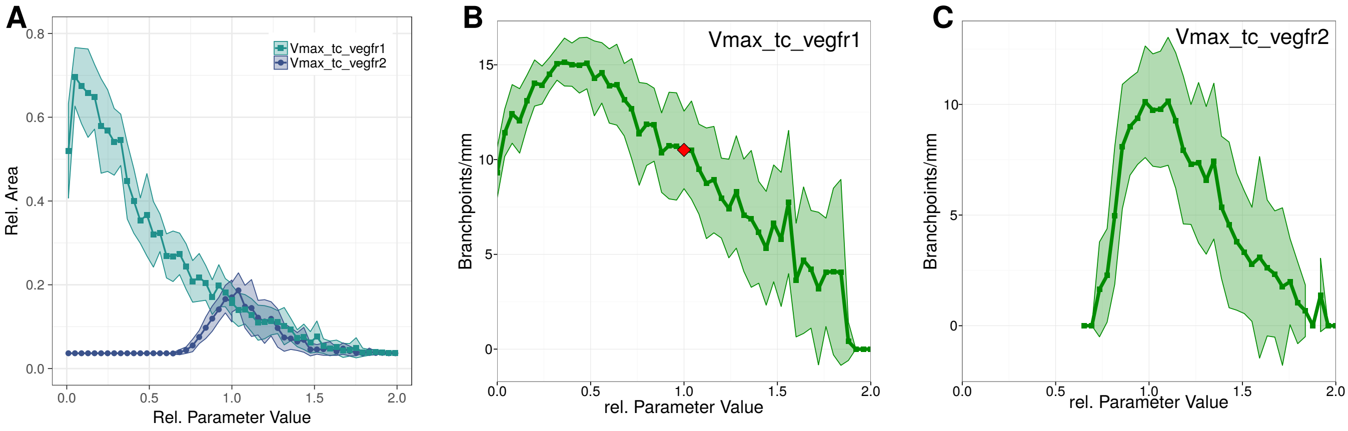

Figure S49: Parameter sensitivity of relative vessel area for $k_{+VEGFR1\_mRNA}$ and $k_{+VEGFR2\_mRNA}$ (A), branch points/mm for $k_{+VEGFR1\_mRNA}$ (B) and branch points/m for $k_{+mVEGFR2\_mRNA}$ (C).

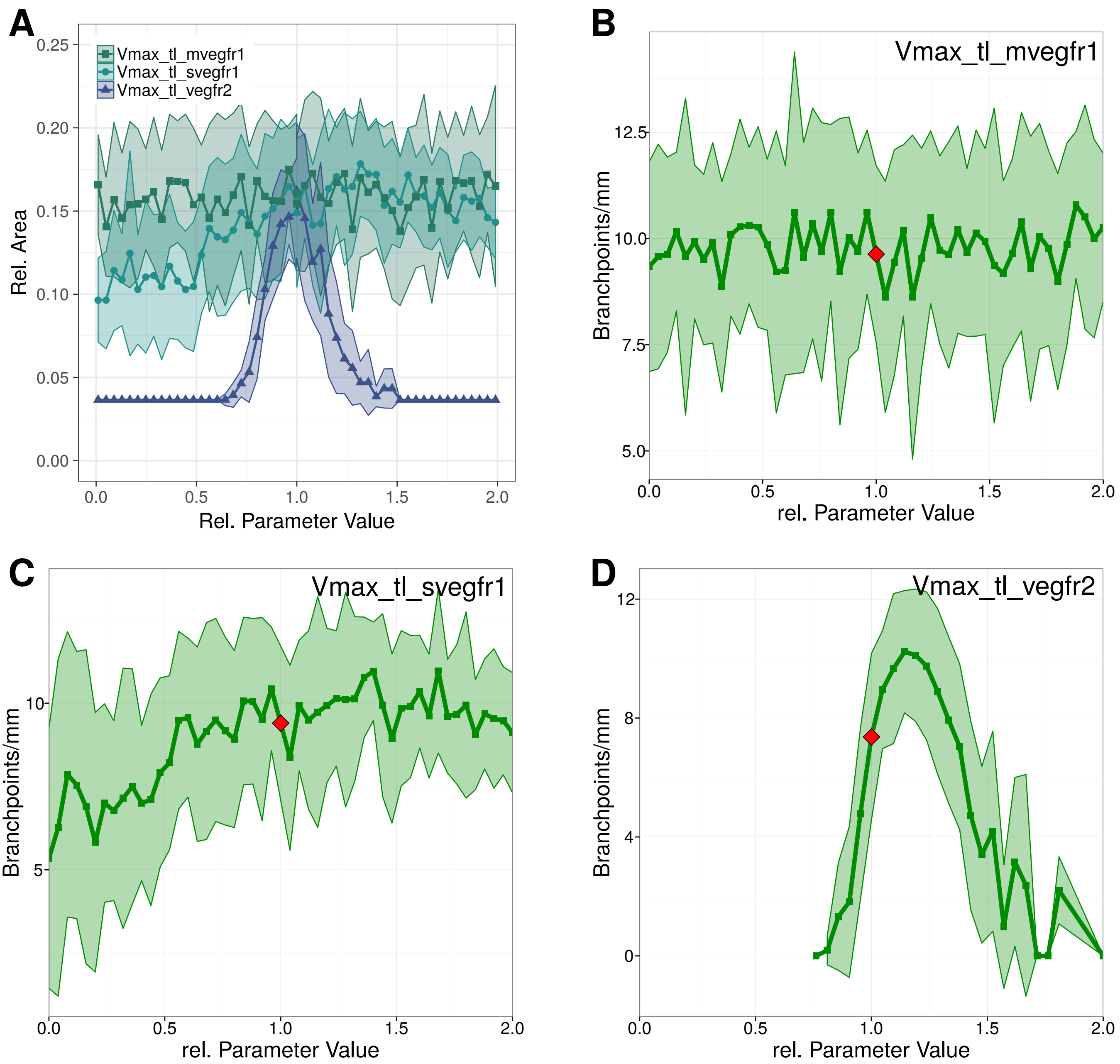

Figure S50: Parameter sensitivity of relative vessel area for $k_{+mVEGFR1}$, $k_{+sVEGFR1}$ and $k_{+VEGFR2}$ (A), branch points/mm for $k_{+mVEGFR1}$ (B), branch points/m for $k_{+sVEGFR1}$ (C), and branch points/m for $k_{+VEGFR2}$ (D).

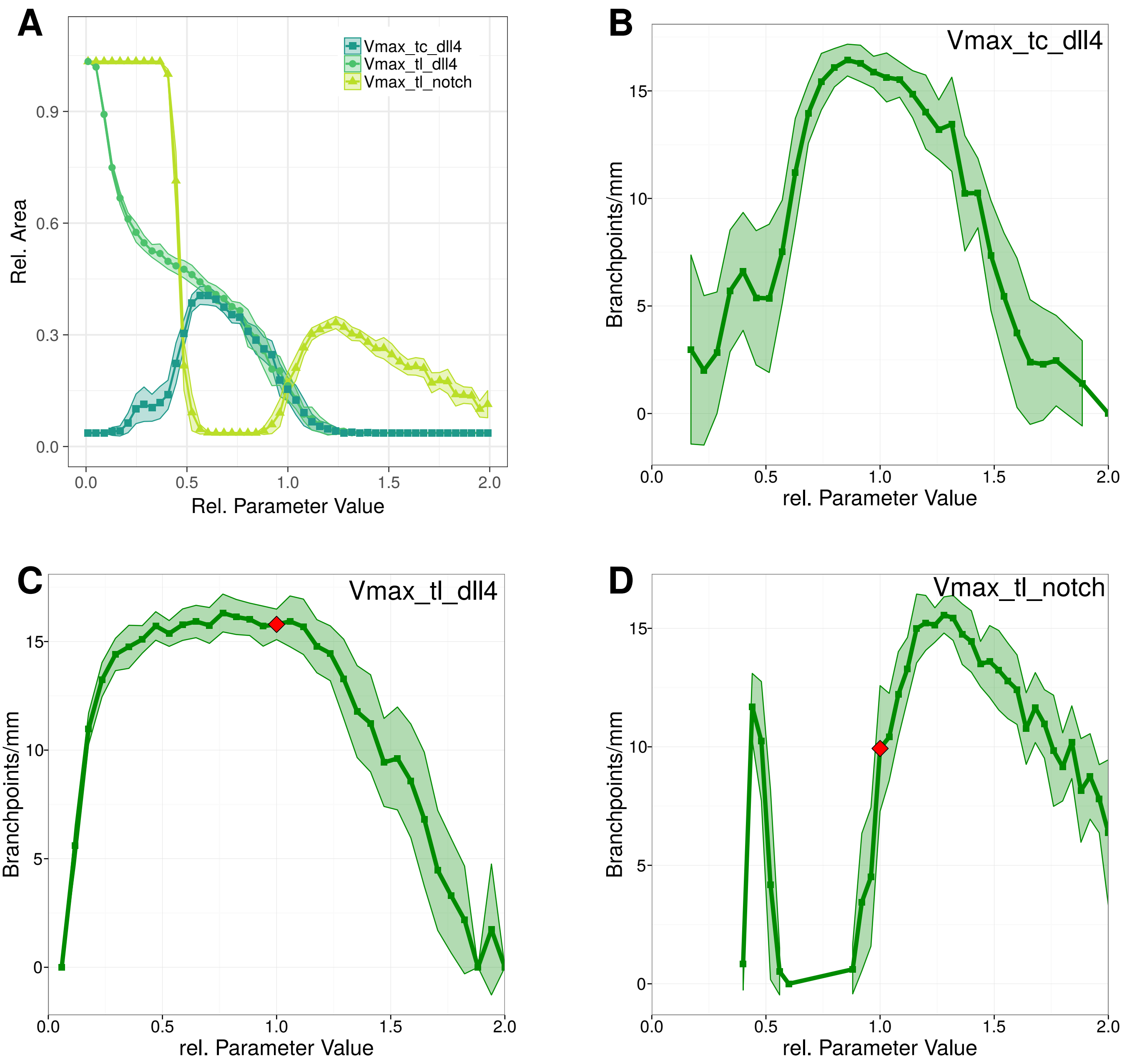

Figure S51: Parameter sensitivity of relative vessel area for k_{+mDll4_mRNA}, k_{+mDll4} and k_{+Notch} (A), branch points/mm for k_{+mDll4_mRNA} (B), branch points/m for k_{+mDll4} (C), and branch points/m for k_{+Notch} (D).

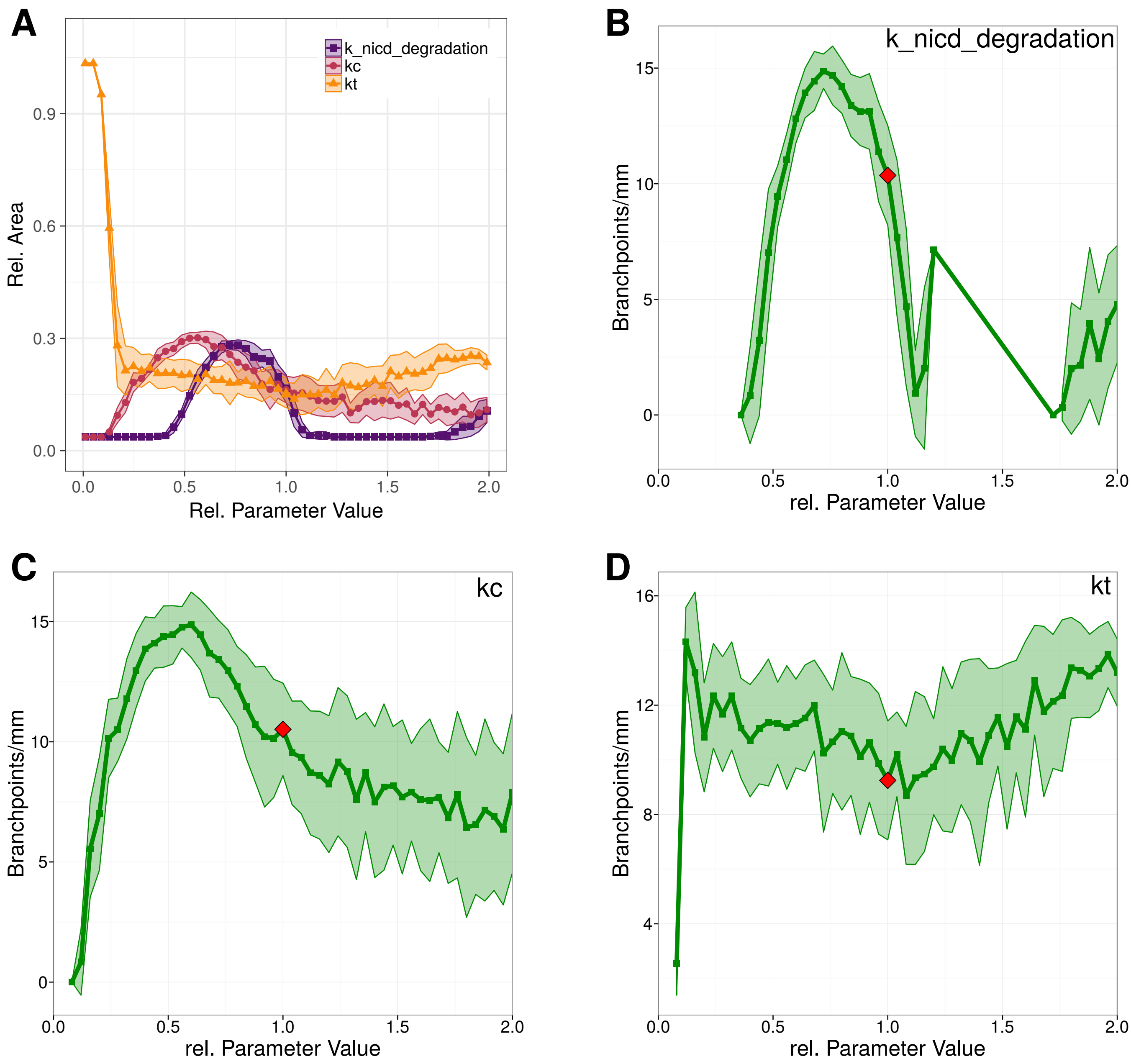

Figure S52: Parameter sensitivity of relative vessel area for k_{-NICD}, k_c and k_t (A), branch points/mm for k_{-NICD} (B), branch points/m for k_c (C), and branch points/m for k_t (D).

REFERENCES

Wang N, Tolić-Nørrelykke IM, Chen J, Mijailovich SM, Butler JP, Fredberg JJ, et al. Cell prestress. I. Stiffness and prestress are closely associated in adherent contractile cells. *American journal of physiology. Cell physiology* **282** (2002) C606–C616. doi:10.1152/ajpcell.00269.2001.

Ubezio B, Blanco RA, Geudens I, Stanchi F, Mathivet T, Jones ML, et al. Synchronization of endothelial Dll4-Notch dynamics switch blood vessels from branching to expansion. *eLife* **5** (2016) 1–32. doi:10.7554/eLife.12167.

Bentley K, Gerhardt H, Bates PA. Agent-based simulation of notch-mediated tip cell selection in angiogenic sprout initialisation. *Journal of Theoretical Biology* **250** (2008) 25–36. doi:10.1016/j.jtbi.2007.09.015.

Mac Gabhann F, Popel AS. Targeting neuropilin-1 to inhibit VEGF signaling in cancer: Comparison of therapeutic approaches. *PLoS Computational Biology* **2** (2006) 1649–1662. doi:10.1371/journal.pcbi.0020180.

Shamloo A, Xu H, Heilshorn S. Mechanisms of Vascular Endothelial Growth Factor-Induced Pathfinding by Endothelial Sprouts in Biomaterials. *Tissue Engineering Part A* **18** (2012) 320–330. doi:10.1089/ten.tea.2011.0323.

Köhn-Luque A, de Back W, Yamaguchi Y, Yoshimura K, Herrero MA, Miura T. Dynamics of VEGF matrix-retention in vascular network patterning. *Physical Biology* **10** (2013) 066007. doi:10.1088/1478-3975/10/6/066007.

Chen RR, Silva EA, Yuen WW, Mooney DJ. Spatio-temporal VEGF and PDGF delivery patterns blood vessel formation and maturation. *Pharmaceutical Research* **24** (2007) 258–264. doi:10.1007/s11095-006-9173-4.

Gabhann FM, Ji JW, Popel AS. Computational model of vascular endothelial growth factor spatial distribution in muscle and pro-angiogenic cell therapy. *PLoS Computational Biology* **2** (2006) 1107–1120. doi:10.1371/journal.pcbi.0020127.

Vempati P, Mac Gabhann F, Popel AS. Quantifying the proteolytic release of extracellular matrix-sequestered VEGF with a computational model. *PLoS ONE* **5** (2010). doi:10.1371/journal.pone.0011860.

Hashambhoy YL, Chappell JC, Peirce SM, Bautch VL, Mac Gabhann F. Computational modeling of interacting VEGF and soluble VEGF receptor concentration gradients. *Frontiers in Physiology* **2 OCT** (2011) 1–12. doi:10.3389/fphys.2011.00062.

Schwanhausser B. Global quantification of mammalian gene expression control. *Nature* **473** (2011) 337–342. doi:10.1038/nature10098.

Carlier A, Geris L, Bentley K, Carmeliet G, Carmeliet P, van Oosterwyck H. MOSAIC: A Multiscale Model of Osteogenesis and Sprouting Angiogenesis with Lateral Inhibition of Endothelial Cells. *PLoS Computational Biology* **8** (2012). doi:10.1371/journal.pcbi.1002724.

Imoukhuede PI, Dokun AO, Annex BH, Popel AS. Endothelial cell-by-cell profiling reveals the temporal dynamics of VEGFR1 and VEGFR2 membrane localization after murine hindlimb ischemia. *American journal of physiology. Heart and circulatory physiology* **304** (2013) H1085–93. doi:10.1152/ajpheart.00514.2012.

Gabhann FM, Popel AS. Interactions of VEGF isoforms with VEGFR-1, VEGFR-2, and neuropilin in vivo: a computational model of human skeletal muscle. *AJP: Heart and Circulatory Physiology* **292** (2006) H459–H474. doi:10.1152/ajpheart.00637.2006.

Boareto M, Jolly MK, Lu M, Onuchic JN, Clementi C, Ben-Jacob E. Jagged-Delta asymmetry in Notch signaling can give rise to a Sender/Receiver hybrid phenotype. *Proceedings of the National Academy of Sciences of the United States of America* **112** (2015) E402–9. doi:10.1073/pnas.1416287112.

Bentley K, Mariggi G, Gerhardt H, Bates PA. Tipping the balance: Robustness of tip cell selection, migration and fusion in angiogenesis. *PLoS Computational Biology* **5** (2009). doi:10.1371/journal.pcbi.1000549.

Venkatraman L, Regan ER, Bentley K. Time to decide? Dynamical analysis predicts partial tip/stalk patterning states arise during angiogenesis. *PLoS ONE* **11** (2016) 1–23. doi:10.1371/journal.pone.0166489.

Schwanhäusser B, Busse D, Li N, Dittmar G, Schuchhardt J, Wolf J, et al. Global quantification of mammalian gene expression control. *Nature* **473** (2011) 337–342. doi:10.1038/nature10098.

Yang E, van Nimwegen E, Zavolan M, Rajewsky N, Schroeder M, Magnasco M, et al. Decay rates of human mRNAs: correlation with functional characteristics and sequence attributes. TL - 13. *Genome research* **13 VN - r** (2003) 1863–1872. doi:10.1101/gr.1272403.

Walpole J, Chappell JC, Cluceru JG, Mac Gabhann F, Bautch VL, Peirce SM. Agent-based model of angiogenesis simulates capillary sprout initiation in multicellular networks. *Integrative biology : quantitative biosciences from nano to macro* **7** (2015) 987–97. doi:10.1039/c5ib00024f.

Wu FTH, Stefanini MO, Mac Gabhann F, Kontos CD, Annex BH, Popel AS. VEGF and soluble VEGF receptor-1 (sFlt-1) distributions in peripheral arterial disease: an in silico model. *American journal of physiology. Heart and circulatory physiology* **298** (2010) H2174–91. doi:10.1152/ajpheart.00365.2009.

Cunningham SA, Tran TM, Arrate MP, Brock TA. Characterization of Vascular Endothelial Cell Growth Factor Interactions with the Kinase Insert Domain-containing. *The Journal of biological chemistry* **274** (2000) 18421–18427.

Sprinzak D, Lakhanpal A, LeBon L, Garcia-Ojalvo J, Elowitz MB. Mutual inactivation of Notch receptors and ligands facilitates developmental patterning. *PLoS Computational Biology* **7** (2011). doi:10.1371/journal.pcbi.1002069.

Harrington LS, Sainson RCA, Williams CK, Taylor JM, Shi W, Li JL, et al. Regulation of multiple angiogenic pathways by Dll4 and Notch in human umbilical vein endothelial cells. *Microvascular Research* **75** (2008) 144–154. doi:10.1016/j.mvr.2007.06.006.

Hiner MC, Rueden CT, Eliceiri KW. ImageJ-MATLAB: A bidirectional framework for scientific image analysis interoperability. *Bioinformatics* **33** (2017) 629–630. doi:10.1093/bioinformatics/btw681.

Arganda-Carreras I, Fernández-González R, Muñoz-Barrutia A, Ortiz-De-Solorzano C. 3D reconstruction of histological sections: Application to mammary gland tissue. *Microscopy Research and Technique* **73** (2010) 1019–1029. doi:10.1002/jemt.20829.
